# New acridone derivatives to target telomerase and oncogenes – an anticancer approach†

**DOI:** 10.1039/d4md00959b

**Published:** 2025-04-17

**Authors:** Tiago J. S. Marques, Diana Salvador, Helena Oliveira, Vanda V. Serra, Nicholas Paradis, Chun Wu, Vera L. M. Silva, Catarina I. V. Ramos

**Affiliations:** a LAQV-REQUIMTE, Department of Chemistry, University of Aveiro 3810-193 Aveiro Portugal c.ramos@ua.pt; b CESAM-Centre for Environmental and Marine Studies, Department of Biology, University of Aveiro 3810-193 Aveiro Portugal; c CICECO, Aveiro Institute of Materials, Department of Chemistry, University of Aveiro 3810-193 Aveiro Portugal; d Centro de Química Estrutural, Institute of Molecular Sciences, Instituto Superior Técnico, Universidade de Lisboa Av. Rovisco Pais 1 1049-001 Lisboa Portugal; e Department of Chemistry and Biochemistry, Rowan University Glassboro New Jersey USA

## Abstract

In this work, two new acridone derivatives, **AcridPy** and **AcridPyMe**, were synthesized, for the first time, aiming to evaluate their potential as quadruplex stabilizers and anticancer agents. **AcridPy** was synthesized through a very straightforward one-pot sequential chemical reaction involving the Heck cross-coupling reaction of (*E*)-3-iodo-2-(4-methoxystyryl)-1-methylquinolin-4(1*H*)-one with a vinyl pyridine followed by *in situ* electrocyclization and oxidation, while the synthesis of **AcridPyMe** involved an additional *N*-methylation of the pyridine ring. Their ability to stabilize G-quadruplex DNA structures, which are associated with the regulation of oncogenes, was assessed using biophysical methods. Both compounds demonstrated significant quadruplex stabilization properties, showing selectivity to G-quadruplexes over duplex DNA. Molecular dynamics simulation experiments supported the preferential binding of **AcridPyMe** to MYC. The cytotoxicity of these derivatives was further evaluated *in vitro* in two distinct pancreatic tumor cell lines, PanC-1 and MIA PaCa-2, the lung tumor A549 cell line, the melanoma A375 cell line, and the immortalized human keratinocyte HaCaT cell line, through the evaluation of cell viability. For PanC-1 and MIA PaCa-2, the cell cycle dynamics and apoptotic cell death along with colocalization were also evaluated. The results revealed that **AcridPyMe** exhibited anticancer activity, correlated with its quadruplex stabilization ability and, although not exclusive, nuclear co-localization was observed. These findings suggest that the newly synthesized cationic acridone is a promising candidate for the development of novel anticancer therapies targeting G-quadruplex structures.

## Abstract 2

G4 stabilization by acridone derivatives to indirectly regulate pancreatic cell growth.

## Graphical abstract

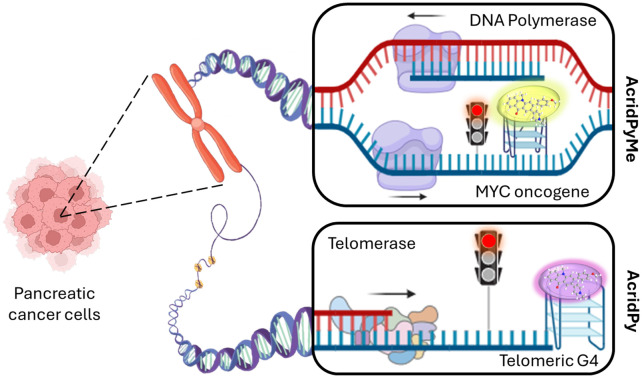

## 1. Introduction

Despite huge efforts in the last decades aiming at cancer eradication, this complex and challenging disease is still growing in a large group of countries, especially in the developed ones, due to the prevalence of unhealthy lifestyles that increase the risk of cancer onset and development.^1,2^ Furthermore, tumor formation can also occur due to the modification of certain molecular mechanisms that can promote the development of this disease.

One of the most challenging cancers is pancreatic cancer, which can include pancreatic ductal adenocarcinoma, a disease responsible for the death of almost 470 000 people in 2022, being ranked as the sixth most deadly cancer in the world, according to the World Health Organization (WHO).^3^

The overexpression of the enzyme telomerase, which is responsible for the elongation of telomeres and is present in about 90% of all human cancers, is one of the molecular mechanisms responsible for the onset of cancer.^4^ Telomerase is constituted by two major subunits – a telomerase reverse transcriptase (TERT) and a telomerase RNA component (TERC) – both associated with important structural proteins (NHP2, NOP10, GAR and dyskerin). Together, this enzymatic complex is responsible for the addition of telomeric repeats TTAGGG in 3′ ends of the telomere.^5^ The expression of this enzyme in most cancers allows the “end-replication problem” to be overcome, leading to a massive growth and uninterrupted cell division scenario of cancer cells.^6,7^

In addition, oncogenes, such as MYC or KRAS, are also closely associated with the initiation of cancer due to their active role in cell division and proliferation. Before undergoing mutation, these genes are present in the human genome in the form of proto-oncogenes, which can participate in some important cellular processes, such as growth and nuclear transcription factors.^8^

**Fig. 1:**
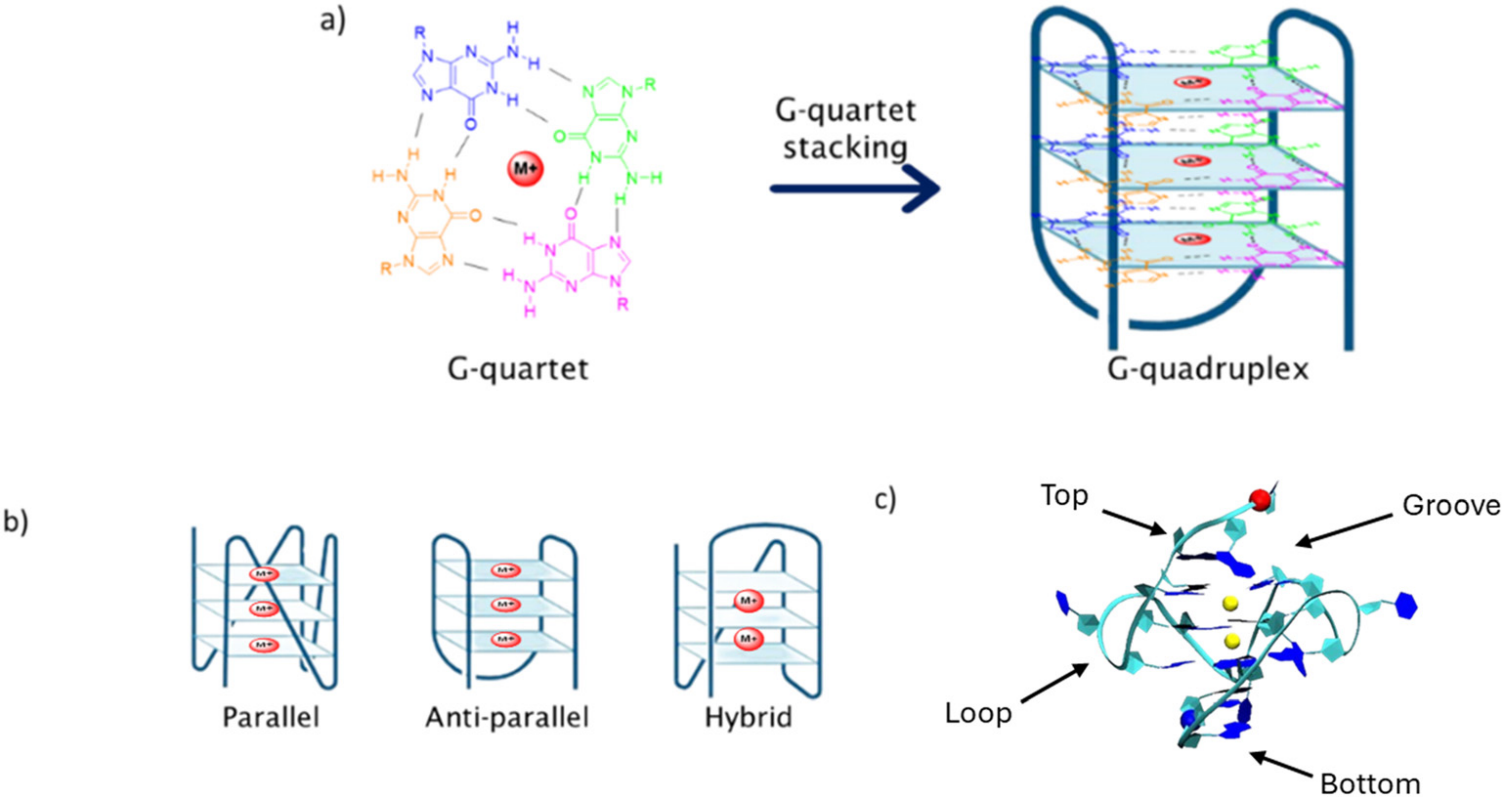
a) Formation of the G-quadruplex structure by stacking of G-quartet units and stabilization by cations (such as Na^+^ and K^+^), represented by the red circle M^+^. Each guanine is represented by a different color. b) Three main structural conformations adopted by G4 structures. c) Four different binding sites of a ligand in the G4 structure.

The presence, in both oncogene and telomeric regions, of repetitive genomic sequences containing high abundance of guanine (G), able to form secondary DNA structures, known as G-quadruplexes (G4s), makes oncogenes and telomeres potential antitumor targets.^9,10^ G4s are assembled by the stacking of G-quartets (Fig. 1a), formed by four guanines, and supported by Hoogsteen bonds between these nitrogen bases, enabling the establishment of unconventional hydrogen bonds with other purines.^11^ These genomic elements are highly associated with the regulation of some important cellular processes, such as translation, processing of mRNA, and replication initialization, among others.^12,13^

G4s are highly polymorphic and can adopt different structural conformations depending on the DNA strand orientation, the presence of different ionic neighborhood and internal or external elements, such as temperature. Thus, G4s can be found in three main conformational arrangements: parallel, anti-parallel or hybrid (Fig. 1b).^14^ Highly abundant cations, such as Na^+^ and K^+^, which play important roles in metabolic processes, are usually involved in G4 stabilization. It is interesting to note that the presence of a different cation will give rise to different G4 structural conformation. For example, for the telomeric G4 DNA d[AG_3_(T_2_AG_3_)_3_], the presence of K^+^ results in the formation of a hybrid topology, while Na^+^ gives rise to an antiparallel conformation (Fig. 1b).^15^

Due to the importance of these secondary genomic structures in cell regulation, the stabilization or unfolding of G4s may help in the enhancement or control of certain processes. This relatively new approach to cancer growth control is focused on the stabilization of G4 structures, therefore preventing the enzymatic activity of telomerase in telomeres or of DNA polymerase II in oncogenes.^16,17^

In the past years, several ligands have been synthesized and evaluated as potential G4 stabilizing agents, with the aim of interrupting telomerase/polymerase activity.^18–22^ These ligands can interact with the G4 structure through different modes: in the upper or in the lower end of the G4 tetrad core, known as end-stacking, the interaction occurring with the G4 backbone, so-called groove or loop binding, and between the tetrads, recognized as intercalation (Fig. 1c). The process of intercalation between tetrads is considered a difficult process, since the structural conformation of the G4 is stable and presents some rigidity. Therefore, this specific type of binding should involve a high amount of energy, thus end-stacking, loop or groove binding modes are considered energetically more favorable.^23^

**Fig. 2:**
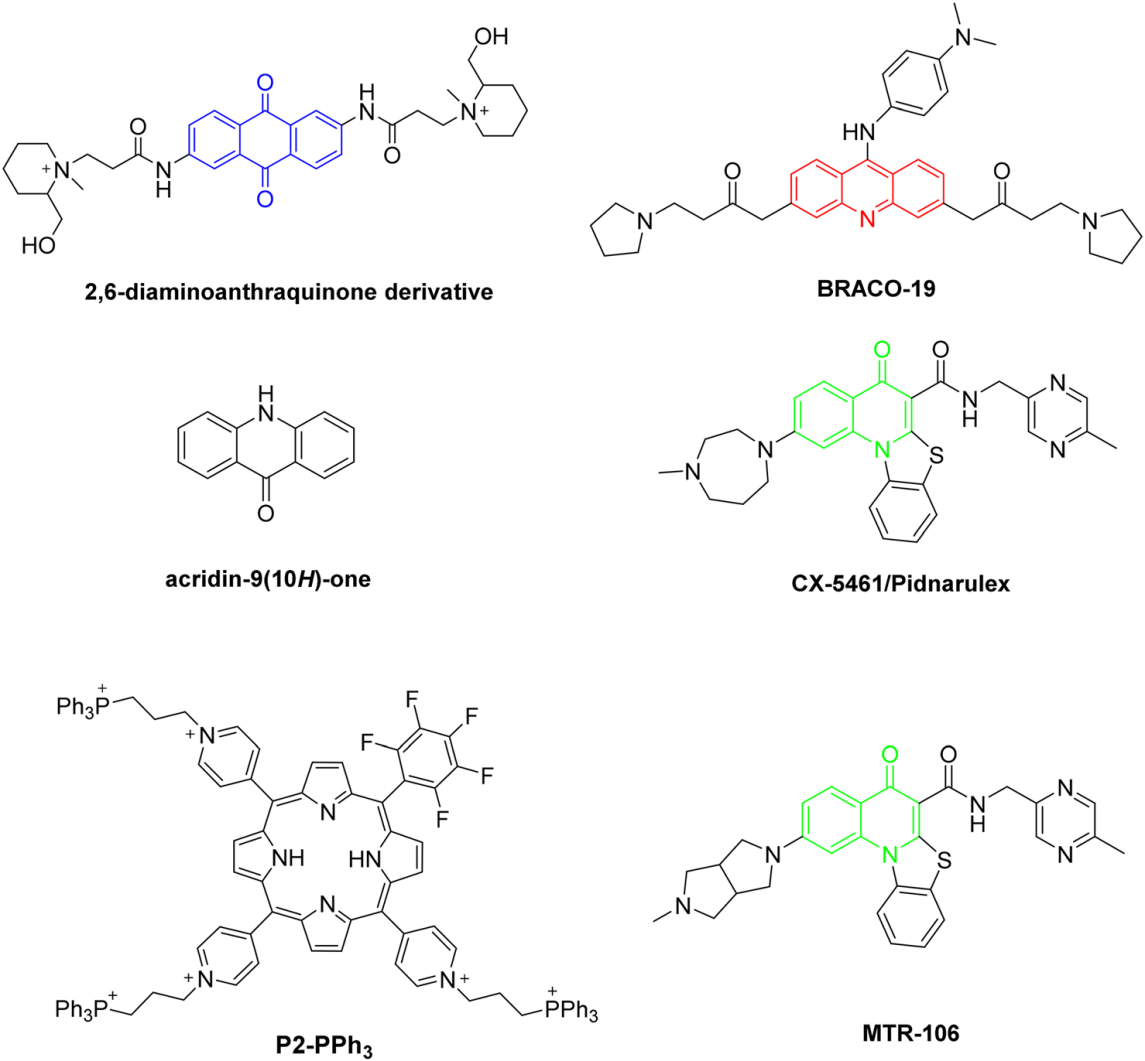
Some examples of G4 stabilizing ligands.

A large number of organic compounds have been evaluated as G4 stabilizers and as potential anticancer drugs, with the 2,6-diaminoanthraquinone derivative (Fig. 2) identified as the first G4-stabilizing organic ligand.^24^Fig. 2 shows examples of other promising ligands studied since then, along with their given/commercial name. Some of these molecules, such as CX-5461, are already in clinical phase trials and present favorable interactions with the G4 DNA and direct correlation with the tumor's growth control.^20,25,26^ Other well-known G4 ligands include large heterocyclic compounds like phthalocyanines and porphyrins, such as P2-PPh_3_. These two families of ligands are widely studied as G4 stabilizers due to their particular planar core, similar to the G-quartet design.^22,27–30^

**Scheme 1:**
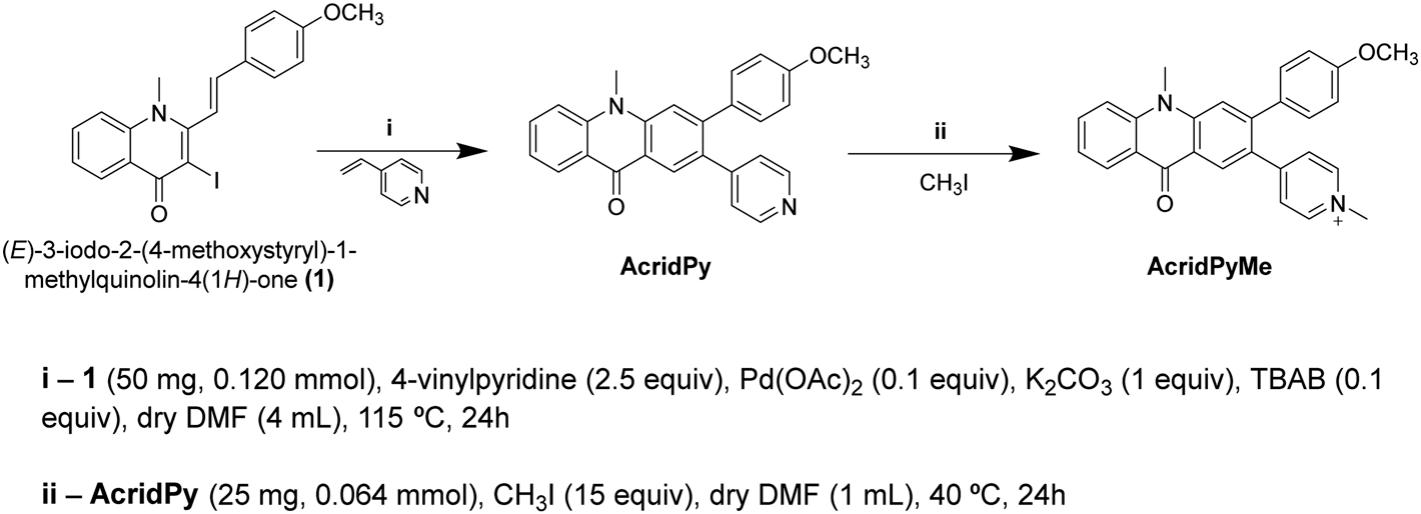
Synthesis steps of both **AcridPy** and **AcridPyMe**.

Among G4 stabilizers, special attention is being focused on acridones. These heterocyclic derivatives are well-known for their diverse biological activities, including antimicrobial, antiviral, and anticancer properties.^31–33^ The planar structure of acridones allows for effective interaction with DNA, making them particularly attractive as candidates for G4 stabilization. Previous studies have shown that certain acridone derivatives can interact with nucleic acids,^34–37^ but there is a limited understanding of their specific interactions with G4 structures and their potential as anticancer agents. By comparing the structures presented in Fig. 2, it is possible to observe a similarity between the acridine core (in red) of BRACO-19 and the central core of the acridones **AcridPy** and **AcridPyMe** (*vide infra*Scheme 1) described herein. In addition, it is interesting to note that several studies indicate that ligands having charged substituents exhibit higher affinity towards G4 than its neutral analogues.^22,38^ This characteristic was also considered in the design of the ligands described in this study.

The present work aims to evaluate the ability of the two novel acridone derivatives, **AcridPy** and **AcridPyMe**, to stabilize G4 DNA structures (G4 Tel, MYC and KRAS) and to identify their potential to be used in anticancer therapies. The interaction between the neutral and cationic acridone derivatives with synthetic DNA structures was assessed through several biophysical and biochemical techniques, including fluorescence spectroscopy, fluorescence intercalator displacement (FID) and circular dichroism (CD).^39–42^ Molecular Dynamics (MD) simulations were also conducted to assess the preferential binding of the ligands to the selected G4 sequences in comparison to a double-stranded sequence (ds26). Cytotoxicity was evaluated in two distinct pancreatic tumor cell lines, PanC-1 and MIA PaCa-2, the lung tumor A549 cell line, the melanoma A375 cell line, and the immortalized human keratinocyte HaCaT cell line, through the evaluation of cell viability. For PanC-1 and MIA PaCa-2, cell cycle analysis, apoptosis, and colocalization by FLIM microscopy were also evaluated.

All together the results demonstrated the potential of the novel cationic acridone **AcridPyMe** to target G4 structures and to be used as a new therapeutic agent against pancreatic cancer cells.

## 2. Results and discussion

### 2.1. Synthesis and characterization of **AcridPy** and **AcridPyMe**

**AcridPy** was obtained starting from the (*E*)-3-iodo-2-(4-methoxystyryl)-1-methylquinolin-4(1*H*)-one precursor *via* the Heck reaction with 4-vinylpyridine using a similar procedure to that reported for the synthesis of (*E*,*E*)-2,3-distyrylquinolin-4(1*H*)-one derivatives (Scheme 1).^43^ However, after the coupling reaction, an *in situ* electrocyclization and oxidation took place affording the acridone derivative as the main reaction product. The obtained compound was then methylated with iodomethane, resulting in the formation of **AcridPyMe**. The structures of these compounds (Scheme 1) were confirmed by ^1^H and ^13^C NMR spectroscopy as well as by mass spectrometry (ESI-MS and ESI-HRMS) (Fig. S1–S6†).

The ^1^H NMR spectrum of **AcridPy** showed two singlets in the aliphatic region, corresponding to the resonance of the protons of the methoxy (–OCH_3_) group, at *δ* = 3.84 ppm, and of the methyl group linked to the nitrogen (–NCH_3_) in the acridone core, at *δ* = 4.04 ppm. In the aromatic region the singlets appearing at *δ* = 8.56 ppm and *δ* = 7.82 ppm were ascribed to the resonance of protons 1 and 4, respectively, from the new fused aromatic ring (please see numbering of both compounds in Fig. S1 and S3†). The other aromatic protons were identified based on their chemical environment and showed the expected splitting pattern. The analysis of the ^13^C NMR spectrum, with the aid of HSQC and HMBC spectra, showed the presence of two characteristic peaks in the aliphatic region, corresponding to the methoxy group (–OCH_3_), at *δ* = 33.2 ppm, and the methyl group (–NCH_3_), *δ* = 54.4 ppm.

The main difference between the ^1^H NMR spectra of **AcridPy** and **AcridPyMe** is the presence of an additional singlet at *δ* = 4.36 ppm due to the resonance of the protons from the extra *N*-methyl group in the pyridinium unit. In both spectra, the remaining peaks show a similar pattern, apart from small deviations caused by the presence of the new positive charge (Fig. S1 and S3†). Also, in the ^13^C NMR spectrum of **AcridPyMe** it was possible to find an extra peak, at *δ* = 46.6 ppm, corresponding to the new *N*-methyl group in the pyridine moiety.

The mass spectrum of **AcridPy** showed a peak at *m*/*z* 393.2, corresponding to its protonated molecular ion [M + H]^+^, while for **AcridPyMe** the peak at *m*/*z* 407.2, corresponding to M^+^, confirmed the presence of the additional methyl group (Fig. S5 and S6†).

**Fig. 3:**
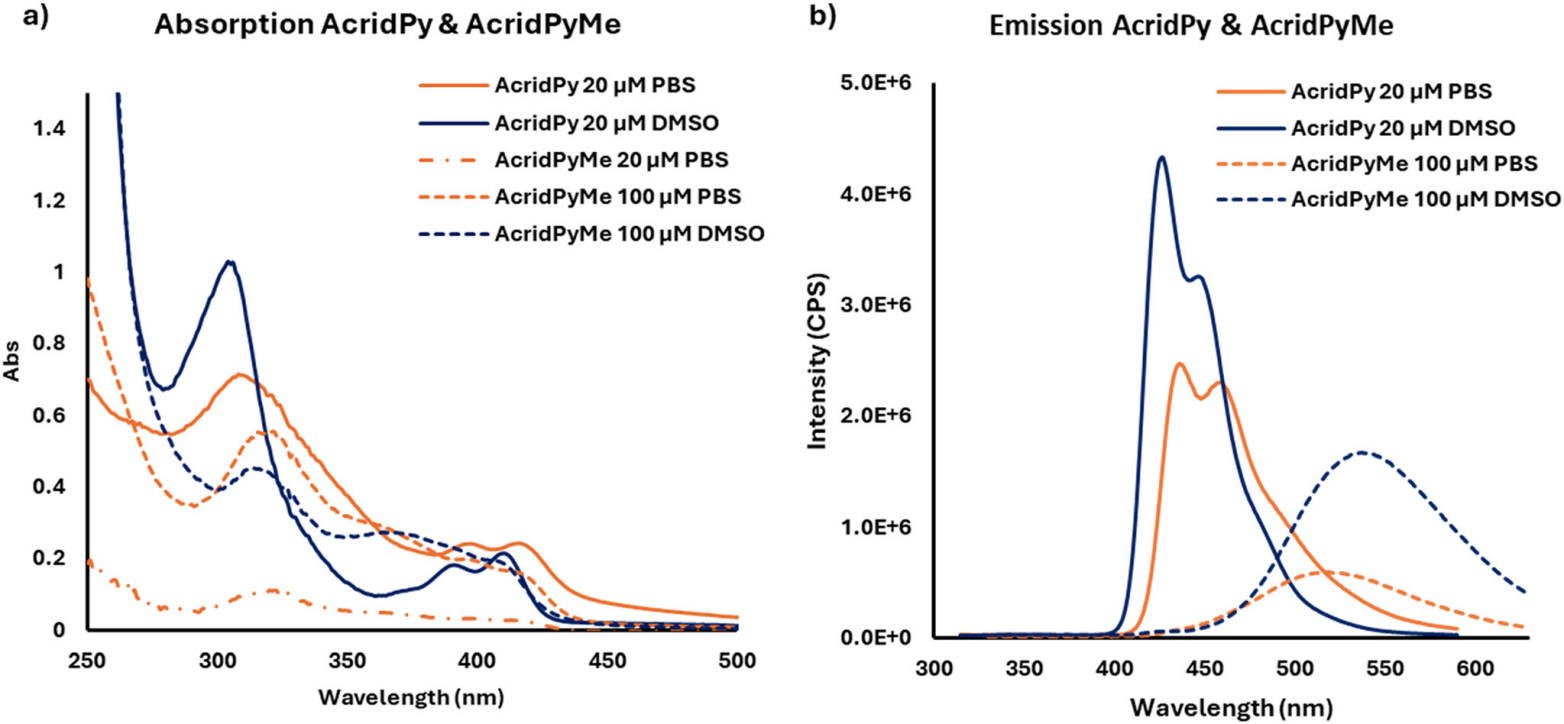
Absorption spectra a) and emission spectra b) of **AcridPy** and **AcridPyMe**.

Regarding the photophysical properties of both ligands, their absorption spectra, recorded in DMSO and PBS, showed an intense band at *ca.* 320 nm, which is characteristic of typical transitions (π → π* or *n* → π*) in aromatic and conjugated molecules. Less intense bands between 380 and 420 nm reflect the presence of heteroatoms in the ligand structures. The emission spectrum of **AcridPy** shows a band with a shoulder in the 400 to 500 nm range, while the cationic counterpart shows a broadband between 430 and 630 nm. In both the absorption and emission spectra, the cationic ligand **AcridPyMe** shows less intense peaks (Fig. 3). It is worth noting that a higher concentration of **AcridPyMe** (100 μM) was required since the absorbance at 20 μM is around 0.1 thus being in the error range of the equipment and compromising the band definition.

**Fig. 4:**
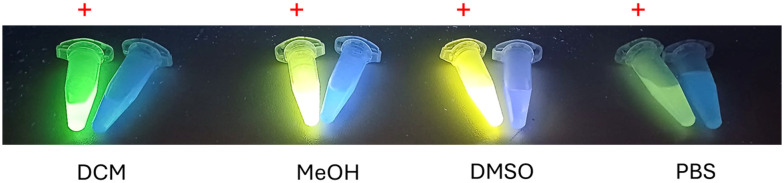
Photographs of **AcridPyMe** (indicated by the red plus sign) and **AcridPy** solutions in dichloromethane (DCM), methanol (MeOH), dimethyl sulfoxide (DMSO) and phosphate-buffered saline (PBS), taken under UV light (∼365 nm).

Considering the absorption and emission spectra of both molecules in DMSO (Fig. 3), a Stokes shift of approximately 120 and 220 nm can be noticed in **AcridPy** and **AcridPyMe**, respectively. This large difference between the maximum absorption wavelength and the emission wavelength presents an advantage for the use of these molecules in biological applications, such as cell imaging and tracing.^44,45^ The fluorescence of both ligands under UV light (∼365 nm) in different solvents is shown in Fig. 4. In PBS, although a considerable decrease of fluorescence was observed, both ligands remained emissive. The fluorescence quantum yield (*Φ*_S_) values in DMSO, determined according to the procedure detailed in the Experimental Section, were 0.210 for **AcridPy** and 0.675 for **AcridPyMe** (see Fig. S10†).

### 2.2. Fluorescence spectroscopy

#### 2.2.1. Fluorescence titrations

Considering the emissive features of **AcridPy** and **AcridPyMe**, their ability to stabilize G4 DNA structures was initially evaluated through fluorescence titrations. This approach is widely used to study interactions between DNA and small ligand molecules, offering several advantages, including high sensitivity, a broad linear concentration range, and selectivity.

Usually, during fluorometric titrations, the occurrence of DNA–ligand interactions can be easily detected by an increase (hyperchromism) or a decrease (hypochromism) in fluorescence intensity. The formation of the ligand–DNA complex can lead to structural compactness in both the drug and/or the DNA, which may result in hypochromism. Furthermore, red (bathochromic effect) or blue shifts (hypsochromic effect) can also occur simultaneously, as a result of changes in the electronic environment. The red shift is indicative of a coupling between the π orbital of the DNA and the π* orbital of the ligand, reducing the π–π* transition energy.^46^ On the other hand, when a ligand binds closely to DNA, the π-electrons in the DNA bases can modify the ligand's electronic transitions, increasing the energy gap between the ligand's ground and excited states which results in a hypsochromic shift (blue shift) in the fluorescence spectrum.^42,47,48^

These titrations also allow the determination of the apparent dissociation constant (*K*_D_), which is indicative of the tendency of the DNA–ligand adduct to dissociate, with lower values of *K*_D_ corresponding to stronger interactions.^49^ In addition, the Hill coefficient (*n*), indicative of the binding cooperativity between the ligand and the G4 DNA, was determined using the Hill saturation model.

In this work, the fluorescence titrations were performed in the presence of G4-Tel, MYC, KRAS and also of the double stranded DNA conformation (ds26) (see oligonucleotide sequences in Table 1) in order to evaluate the selectivity of the new acridones **AcridPy** and **AcridPyMe** to G4 structures. Since a large percentage of DNA in the cellular environment exists in the double-stranded form, ligands must exhibit higher affinity and selectivity for G4 DNA structures. Otherwise, their availability to interact with G4 structures will be limited by their interactions with dsDNA.

**Table 1:** Oligonucleotide sequences used in the evaluation of G4–acridone interactions

Abbreviation	Oligonucleotide sequence	Incidence
ds26	5′-CAA TCG GAT CGA ATT CGA TCC GAT TG-3′	Duplex DNA
MYC	5′-TGA GGG TGG GTA GGG TGG GTA A-3′	Oncogene promoter
KRAS	5′-AGG GCG GTG TGG GAA GAG GGA AGA GGG GGA GG-3′	Oncogene promoter
G4 Tel	5′-AGG GTT AGG GTTAGG GTT AGGG-3′	Telomeric regions

For the sake of simplicity, the hybrid conformation of telomeric G4 obtained in the presence of K^+^ ions will be represented as G4 Tel and the antiparallel conformation obtained when Na^+^ ions are present will be represented as G4 Tel in Na^+^.

**Fig. 5:**
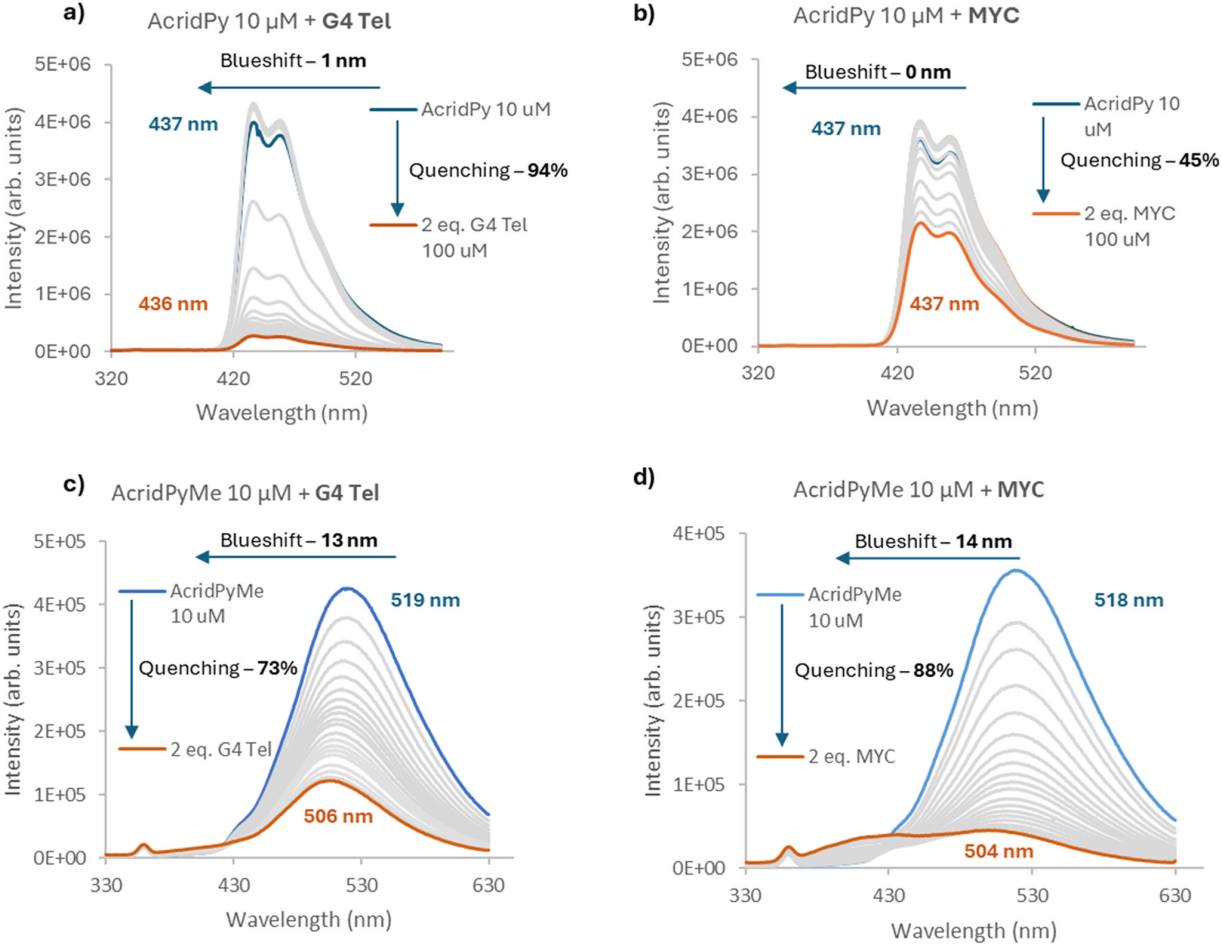
Fluorescence titration spectra of **AcridPy** with a) G4 Tel, b) MYC, and **AcridPyMe** with c) G4 Tel and d) MYC.

The spectra obtained from the titrations of **AcridPy** and **AcridPyMe** with G4 Tel and MYC are shown in Fig. 5. The spectra obtained for the telomeric G4 Tel in Na^+^, KRAS, and ds26 are presented, as ESI,† in Fig. S11 and S12, respectively.

All titrations resulted in a quenching of the fluorescence intensity, though this quenching had different values for the various oligonucleotides. **AcridPy** showed a more pronounced quenching when interacting with G4 Tel (∼94%), with a slight quenching being observed for MYC (∼45%). In the case of **AcridPyMe** although a considerable quenching was observed with G4 Tel (∼73%), a higher quenching was observed towards MYC (∼88%). As can be seen in Fig. 5c and d, S12b (KRAS) and S12c (ds26), along with the above-mentioned quenching effect, pronounced blue shifts, from 13 to 19 nm, were also observed in the titrations of the cationic derivative **AcridPyMe** with the sequences able to form G4 structures. The occurrence of a blue shift can be correlated with the ligand–DNA binding strength, where larger shifts suggest stronger binding, whereas smaller shifts may indicate weaker or more distant binding.

From the data obtained, it is clear that, when compared to **AcridPy**, the cationic acridone, **AcridPyMe**, presents considerable high affinity and selectivity for the G4 structures, the higher blue shifts observed with the sequences able to form G4 structures, pointing to the occurrence of strong interactions. The obtained quenching percentages, spectroscopic shifts and dissociation constants are summarized in Table 2.

**Table 2:** Quenching values and apparent equilibrium dissociation constants of **AcridPy** and **AcridPyMe** obtained by fluorescence titrations

		G4 Tel	G4 Tel Na^+^	MYC	KRAS	ds26
**AcridPy**	Quenching (%)	**94/91**	87/43	45/42	86/83	93/90
	Blueshift (nm)	**1**	1	0	2	1
	*K*_D_ (μM) ± error	**2.58**	3.45	10.85	5.51	3.73
		**0.03**	0.05	0.30	0.079	0.02
	Hill coefficient (*n*)	**7.83**	4.66	4.68	8.07	15.27
**AcridPyMe**	Quenching (%)	73/47	72/41	**88/62**	69/43	43/17
	Blueshift (nm)	13	17	**19**	3	10
	*K*_D_ (μM) ± error	3.30	2.08	**1.86**	9.62	14.95
		0.11	0.07	**0.04**	0.70	1.00
	Hill coefficient (*n*)	0.98	0.80	**1.33**	0.97	0.98

The **AcridPy** derivative showed almost no selectivity towards G4 structures, since it also showed a great binding affinity towards duplex DNA (ds26), with a quenching of ∼93%, a slight blueshift of 1 nm and a dissociation constant (*K*_D_) of 3.73 μM (see Fig. S11c†). In contrast, **AcridPyMe** is clearly more selective towards G4, especially for the MYC oncogene, with a *K*_D_ of 1.86 μM, eight-fold lower than the *K*_D_ of ds26 (*K*_D_ = 14.95 μM), therefore revealing a promising and important feature of selectivity of this ligand. Regarding the Hill coefficient (*n*), **AcridPy** showed all coefficients higher than 1, indicating a positive cooperativity between the neutral ligand and the selected DNA structures. On the other hand, **AcridPyMe** presented only a value of *n* higher than 1 when interacting with MYC structures, showing values lower than 1 when interacting with the remaining DNA sequences, which is indicative of a negative cooperativity.^50^

Titrations with PBS and Tris-NaCl buffer solutions (blank experiments) were also performed with both ligands to determine the contribution of buffer to the fluorescence quenching observed in each titration (please see Fig. S13†). The observed quenching values in the titrations with PBS solution were 3% and 26% for **AcridPy** and **AcridPyMe**, respectively. In the case of Tris-NaCl, values of 44% for **AcridPy** and 31% for **AcridPyMe** were observed, showing a higher contribution of this buffer to fluorescence quenching.

**Fig. 6:**
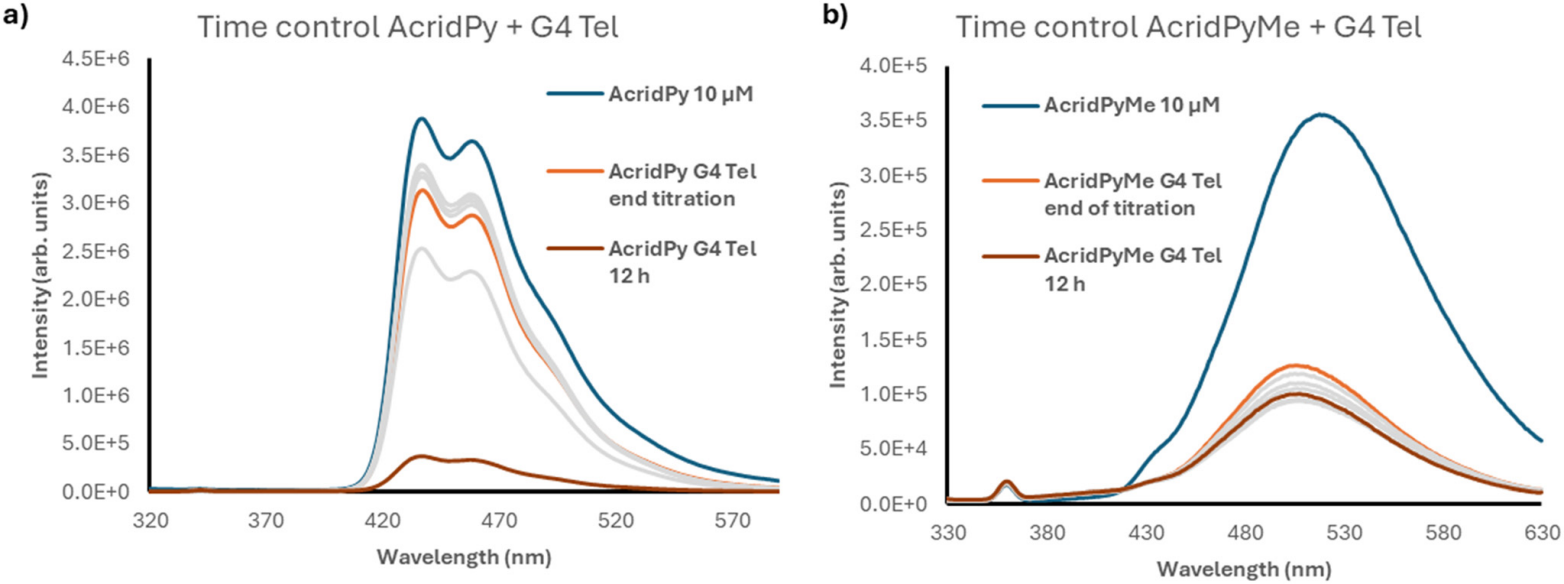
Time control spectra of G4 Tel with 10 μM of a) **AcridPy** and b) **AcridPyMe**, respectively.

To better understand the data obtained and to evaluate the influence of time on the ligand–G4 DNA interaction, the fluorescence intensity of the G4–ligand adducts formed by addition of 2 equiv. of different oligonucleotides to both compounds was monitored for 12 h (see Fig. 6 for G4 Tel and Fig. S14† for the other oligonucleotides). It is interesting to note that **AcridPyMe** had no or little variation in fluorescence intensity after 12 h of interaction, revealing that all interactions occur almost immediately upon addition of the ligand. On the other hand, the results obtained for **AcridPy** point to a slower interaction, with a significant fluorescence intensity decrease after a period of 12 h of interaction with the oligonucleotides.

As depicted in Fig. 6, when comparing the fluorescence intensity of **AcridPyMe** in the end of the titration and after 12 h of interaction with G4 Tel, a quenching of 20% in the fluorescence occurred. For **AcridPy**, after 12 h of interaction, a decrease of about 4 times in the fluorescence intensity, corresponding to a quenching percentage of 88.3%, was observed.

These interesting differences observed in time-controlled measurements may be explained by the presence of the positive charge in **AcridPyMe**, favoring electrostatic interactions with the negative net charge of the oligonucleotide backbone. This correlation between the efficiency of the binding and the presence of positive charges was previously described in porphyrins, where multicharged porphyrins have shown a higher stabilization of the DNA adduct, when compared with the neutral ligands.^22^

#### 2.2.2. G4-FID assay

##### 2.2.2.1. Thiazole Orange

Thiazole orange (TO) is a well-known end-stacking binding probe that only emits fluorescence when bound to DNA structures.^51^ G4-FID assay was performed to assess the ability of both ligands to displace the TO probe from the G4 DNA structures and to obtain the DC_50_ value, which corresponds to the concentration of the ligand necessary to displace TO by 50% from the preformed TO–DNA adduct. The displacement can be followed by adding increasing amounts of the ligand to the preformed TO–DNA solution. The obtained DC_50_ reflects the affinity of ligands to the different DNA structures, with a lower concentration of the ligand being indicative of higher affinity of the ligand to DNA structures.

Surprisingly, no significant alteration in fluorescence intensity occurred when **AcridPy** or **AcridPyMe** was added to the adduct solutions (Fig. S15†), indicating that TO has not been displaced from the preformed TO–DNA adducts. This might indicate that both ligands preferentially bind DNA through a process different from end-stacking.

Considering the results obtained from the fluorescence titrations, which indicated the occurrence of an interaction between the ligands and G4, we postulate that an adduct comprising TO–DNA–Ligand is probably being formed. This interaction may occur through ligand binding at alternative sites on the G4 structures, possibly through groove or loop binding. It is interesting to note that a decrease in fluorescence intensity is expected when electrostatic, hydrogen bonding or hydrophobic interaction with the sugar-phosphate backbone occurs, as in the case of groove.^42^

To evaluate the occurrence of a process of groove binding, FID was also performed using the fluorescent probe Hoechst 33258 in substitution of TO, due to its proven groove binding ability.^52^

##### 2.2.2.2. Hoechst 33258

In this experiment it was possible to observe the displacement of Hoechst 33258 from the Hoechst–G4 adduct, by **AcridPyMe**, which was shown by the decrease in the fluorescence of the adduct, with a quenching value of nearly 35% (Fig. S16†). Interestingly, a red shift is seen as the titration is performed, indicating that the **AcridPyMe**–MYC adduct presents a higher maximum wavelength than the Hoechst–MYC adduct. However, after a few more ligand additions, the displacement could not be tracked due to the appearance of a new species in the spectra that presented a larger red shift, probably corresponding to an excess of free ligand that interferes with the fluorescence of the DNA adduct. This displacement may indicate a possible competition between **AcridPyMe** and Hoechst for the groove binding of the G4 adduct.

### 2.3. Circular dichroism

#### 2.3.1. CD spectra

As reported in the introduction, G4 presents conformational characteristics that depend on the type of cation present in solution. For example, in the case of G4-Tel, K^+^ enhances the hybrid topology, while Na^+^ favors the antiparallel topology.

The G4 formed in oncogene MYC presents a parallel conformation, characterized by a particular CD spectrum, with a positive and a negative band at about 260 and 240 nm, respectively. On the other hand, the antiparallel conformation, in the case of G4 Tel Na^+^, displays a positive band at 295 nm and a negative band at 260 nm, whilst hybrid structures present two positive bands at 295 and 260 nm and a negative one at 245 nm.^53^

CD titrations were employed to evaluate the ability of ligands to bind and/or induce conformational changes in the selected G4 structures (G4 Tel, G4 Tel Na^+^, and MYC). The spectra were recorded in the absence of ligands and in the presence of increasing concentrations of both ligands, as can be seen in Fig. S17† (G4 Tel and MYC) and in Fig. S18† (G4 Tel Na^+^). The titrations were performed within a range of 0 to 24 μM concentration, corresponding to 0 to 6 molar equivalents of the ligand when compared to the 4 μM of DNA solutions.

The results show that the CD spectra of all the G4 oligonucleotides did not show significant changes in the presence of either ligand, revealing that both can interact with each DNA sequence without damaging the established conformation. The fact that none of the ligands present the ability to change the established conformation of the chosen G4 DNA in the range of concentrations studied also points that the characteristic conformation of the quadruplex is not compromised by the presence of the ligands.

#### 2.3.2. CD melting experiments

The effect of the presence of the ligands in the thermal stability of the selected G4 oligonucleotides was evaluated using CD melting assays. By promoting the thermal denaturation of these sequences in the absence and in the presence of both ligands, a positive difference in the melting temperature (*T*_m_) may indicate proper stabilization of these structures due to the interaction with the synthesized ligands. Alongside the previous CD titrations, and after selecting the most promising ligand–G4 sequence pairs, the thermal stability of the selected G4 sequences in the absence and in the presence of **AcridPy** or **AcridPyMe** was evaluated by CD melting experiments.

**Fig. 7:**
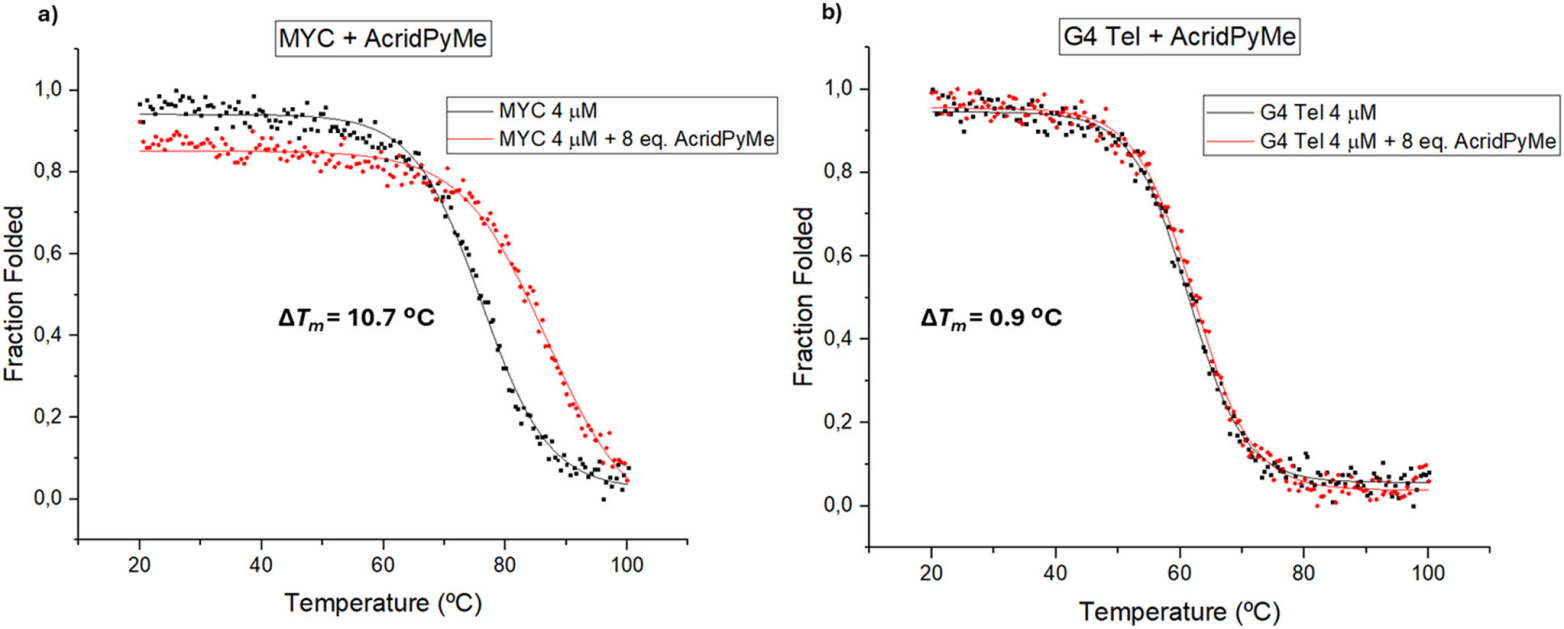
CD melting spectra of a) MYC and b) G4 Tel in the absence and presence of increased concentrations of **AcridPyMe**.

The ellipticity of these measurements was registered at different wavelengths, according to the topology of G4: 290 nm for parallel topology (MYC) and 295 nm for hybrid structures (G4 Tel). In Fig. 7, the obtained melting spectra of MYC and G4 Tel in the absence or presence of **AcridPyMe** are presented.

The melting temperature (*T*_m_) obtained for MYC and for G4 Tel in the absence of ligand was 76.1 °C and 61.4 °C, respectively. An excess of ligand (8 equiv.) was added to ensure that all possible interactions with the G4 structures took place. After incubation with 8 equiv. of the ligands, the *T*_m_ of the MYC–**AcridPyMe** adduct increased by 10.7 °C to 86.8 °C, while G4 Tel–**AcridPyMe** showed a less expressive increment of 0.9 °C, giving a *T*_m_ of 62.3 °C. These results are in line with the earlier data obtained using fluorescence spectroscopy, confirming the binding ability and higher affinity of **AcridPyMe** to MYC structures.

On the other hand, the presence of **AcridPy** in the MYC structure with formation of the MYC–**AcridPy** adduct resulted in an increase in the *T*_m_ of 6.2 °C and a slight increase of 1.7 °C in the case of the adduct G4 Tel–**AcridPy** (Fig. S19†). These results point to the lower affinity of **AcridPy** for G4 structures and are again in line with previous data from fluorescence titrations, described in section 3.2.1. These obtained data are summarized in Table 3.

**Table 3:** CD melting data of both ligands with two different nucleotides (MYC and G4 Tel)

	MYC			G4 Tel		
	*T*_m_i__	*T*_m_f__	Δ*T*_m_	*T*_m_i__	*T*_m_f__	Δ*T*_m_
**AcridPy**	76.3	82.5	**6.2**	61.4	63.1	**1.7**
**AcridPyMe**	76.1	86.8	**10.7**	61.4	62.3	**0.9**

Given the proven stabilization of G4 structures in the presence of the ligands, we decided to complement our studies by performing molecular dynamics to identify the preferential binding modes between both partners, as well as to conduct *in vitro* assays with two different pancreatic tumor cell lines (see sections 2.4 and 2.5).

### 2.4. Molecular dynamics of **AcridPy** and **AcridPyMe**

#### 2.4.1. RMSD and RMSF analysis

The receptor RMSD (R-RMSD) of the nucleic phosphate backbone was calculated for each nucleic acid structure trajectory. The R-RMSD plots were generated (Fig. S20†) and the average values were calculated over the last 50 ns of trajectory time (Table S3†). An R-RMSD value (≤3 Å) is defined to indicate nucleic acid structure stability. In **AcridPy**, MYC, KRAS and G4 Tel systems converged within 50 ns (MYC = 2.7 Å; KRAS = 2.4 Å; G4 Tel = 2.3 Å); in contrast, the ds26 system converged much later, within 400 ns (ds26 = 3.5 Å). In **AcridPyMe**, MYC, KRAS and G4 Tel systems converged within 50 ns (MYC = 2.7 Å; KRAS = 3.2 Å; G4 Tel = 2.3 Å), and the ds26 system converged within 400 ns (ds26 = 3.6 Å). The difference in R-RMSD between **AcridPy** and **AcridPyMe** for MYC (0.0 Å), G4 Tel (0.0 Å) and ds26 (0.1 Å) is considered negligible, whereas that of KRAS (0.8 Å) is more pronounced, but remains below the R-RMSD values indicative of destabilization (R-RMSD > 3.0 Å). The **AcridPyMe**–MYC, **AcridPy**–MYC, **AcridPyMe**–G4 Tel, **AcridPy**–G4 Tel and **AcridPy**–KRAS systems were quite stable, whereas the **AcridPyMe**–KRAS, **AcridPy**–ds26 and **AcridPyMe**–ds26 systems were slightly less stable. All systems were relatively stable and reached convergence.

Additionally, the Ligand-RMSD (L-RMSD) of **AcridPy** and **AcridPyMe** was calculated to check for ligand binding convergence, stability and length of duration for particular binding poses. Similarly, L-RMSD plots were generated (Fig. S20†) and their average values for the last 50 ns of trajectory data were tabulated (Table 4).

R-RMSD of the nucleic acid phosphate backbone, L-RMSD of the ligand heavy atoms and atom contacts of **AcridPy**/**AcridPyMe** for each nucleic acid system averaged across the last 100 ns of trajectory time

**Table:** 

R-RMSD				
	MYC	G4 Tel	KRAS	ds26
**AcridPy**	2.7	2.2	2.4	3.5
**AcridPyMe**	2.7	2.3	3.2	3.6

**Table:** 

L-RMSD				
	MYC	G4 Tel	KRAS	ds26
**AcridPy**	40.7	34.9	40.3	80.8
**AcridPyMe**	50.5	37.7	51.2	53.8

**Table:** 

Atom contacts				
	MYC	G4 Tel	KRAS	ds26
**AcridPy**	16.5	29.6	12.8	12.8
**AcridPyMe**	41.1	15.5	20.6	13.0

For **AcridPy**, the L-RMSD convergence was achieved at different time points for MYC (>220 ns), G4 Tel (<50 to ∼300 ns, and >420 ns), KRAS (>260 ns) and ds26 (>180 ns). For **AcridPyMe**, the L-RMSD convergence was similar for MYC (>220 ns), G4 Tel (<50 to ∼300 ns, and >420 ns). KRAS (>260 ns) and ds26 (>50 ns). Within the last 100 ns of simulation time, the L-RMSD values for **AcridPy** and **AcridPyMe** were generally consistent for G4 Tel (34.9 Å and 37.7 Å), but not for MYC (40.7 Å and 50.5 Å), KRAS (40.3 Å and 51.2 Å) or ds26 (80.8 Å and 53.8 Å). The much higher **AcridPy** and **AcridPyMe** L-RMSD values for ds26 systems than the G4 systems are apparent here. Please note that the high L-RMSD values relative to the R-RMSD values are due to the 10 Å ligand displacement distance from the nucleic acid structures at the beginning of the simulation to allow for global ligand sampling. Some interruptions in the L-RMSD plot for certain systems indicate switching of ligand binding poses. For instance, **AcridPy**–MYC exhibits two periods of L-RMSD change (0 to ∼60 ns and ∼130 to ∼220 ns); **AcridPy**–MYC exhibits one period of L-RMSD change (∼210 ns); **AcridPyMe**–G4 Tel demonstrates a large L-RMSD disturbance late into the simulation before relaxing (∼300 ns to ∼410 ns). Additionally, the global ligand sampling for **AcridPy** and **AcridPyMe** for the KRAS system converges much later relative to the MYC, G4 Tel and ds26 systems (>200 ns), suggesting that these ligands have a longer time to binding to KRAS. The binding modes for **AcridPy** and **AcridPyMe** to each nucleic acid structure will be examined.

#### 2.4.2. Atomic interaction analysis

To elucidate the preferentiality of **AcridPy** and **AcridPyMe** binding to each nucleic acid system, DNA–ligand trajectory snapshot and ligand clustering analyses were conducted for each MD system to analyze their binding poses. The last snapshot structures of **AcridPy** and **AcridPyMe** binding to MYC, G4 Tel, KRAS and ds26 nucleic acid structures are shown in the appendix chapter (Fig. S22†).

**Fig. 8:**
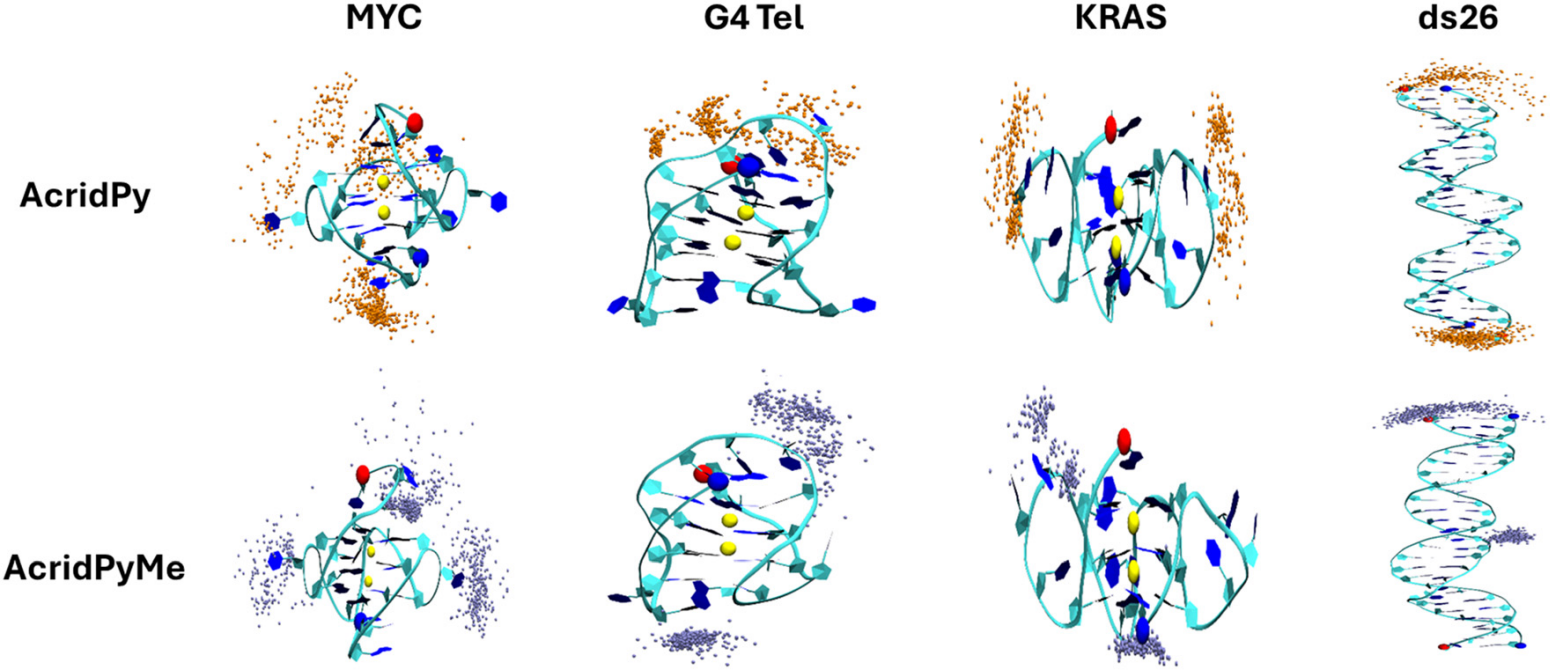
Ligand clustering analysis of **AcridPy** (orange beads) and **AcridPyMe** (ice blue beads) for MYC, G4 Tel, KRAS and ds26 nucleic acid structures. Each bead represents 1 ns snapshot of simulation time of the C1 ligand atom, within 5 Å of the nucleic acid structure, to simplify the clustering viewpoint.

The distribution of **AcridPy** and **AcridPyMe** binding to each nucleic structure is shown in Fig. 8.

Generally, different binding modes were observed between **AcridPy** and **AcridPyMe** for each nucleic acid system. In MYC, **AcridPy** showed two binding cluster nodes: a dense cluster at the bottom G-tetrad layer and a much looser cluster at both the top G4 layer and 3rd groove pocket closer towards the 3′ terminus; in contrast, **AcridPyMe** exhibited three distinct binding clusters at the top G4 layer, the first loop and the 3rd groove pocket. In G4 Tel, **AcridPy** exhibited a single loose cluster at the top G4 layer, whereas **AcridPyMe** exhibited two denser clusters at the top and bottom G4 layers. In KRAS, **AcridPy** and **AcridPyMe** both exhibited two binding clusters, one located near the first groove region closest to the 5′ termini; however, the second cluster was located at the 3rd groove region (**AcridPy**) or the bottom G4 layer (**AcridPyMe**). Lastly in ds26, **AcridPy** exhibits two binding clusters at both termini of ds26, whereas **AcridPyMe** exhibits two binding clusters at one of the termini and another cluster at a groove region.

Atom contact analysis was also performed to provide additional insight into the binding preferences of the compounds to each nucleic acid structure. The atom contact plots and average values of the total contacts between **AcridPy**/**AcridPyMe** and nucleic acid structure over the last 100 ns of simulation time were both generated (Fig. S21† and Table 4). For MYC, **AcridPyMe** exhibited greater contacts (41) compared to **AcridPy** (17). For G4 Tel, the reverse is true for **AcridPy** (30) compared to **AcridPyMe** (16). For KRAS, **AcridPyMe** demonstrated greater contacts (21) than **AcridPy** (13). Lastly, for ds26, **AcridPy** and **AcridPyMe** exhibited the same contact number (13). Conversely, **AcridPyMe** made most contacts with KRAS (69), followed by ds26 (43), MYC (38) and G4 Tel (22). Hence, **AcridPy** and **AcridPyMe** generally engage in more binding with G4s rather than ds26. Combined with the ligand clustering analysis, the additional methyl moiety on **AcridPyMe** seems to confirm the difference in the ligand binding modes compared to **AcridPy** for MYC, G4 Tel, KRAS and ds26 systems.

### 2.5. Biological assays

#### 2.5.1. MTT viability assay

**Fig. 9:**
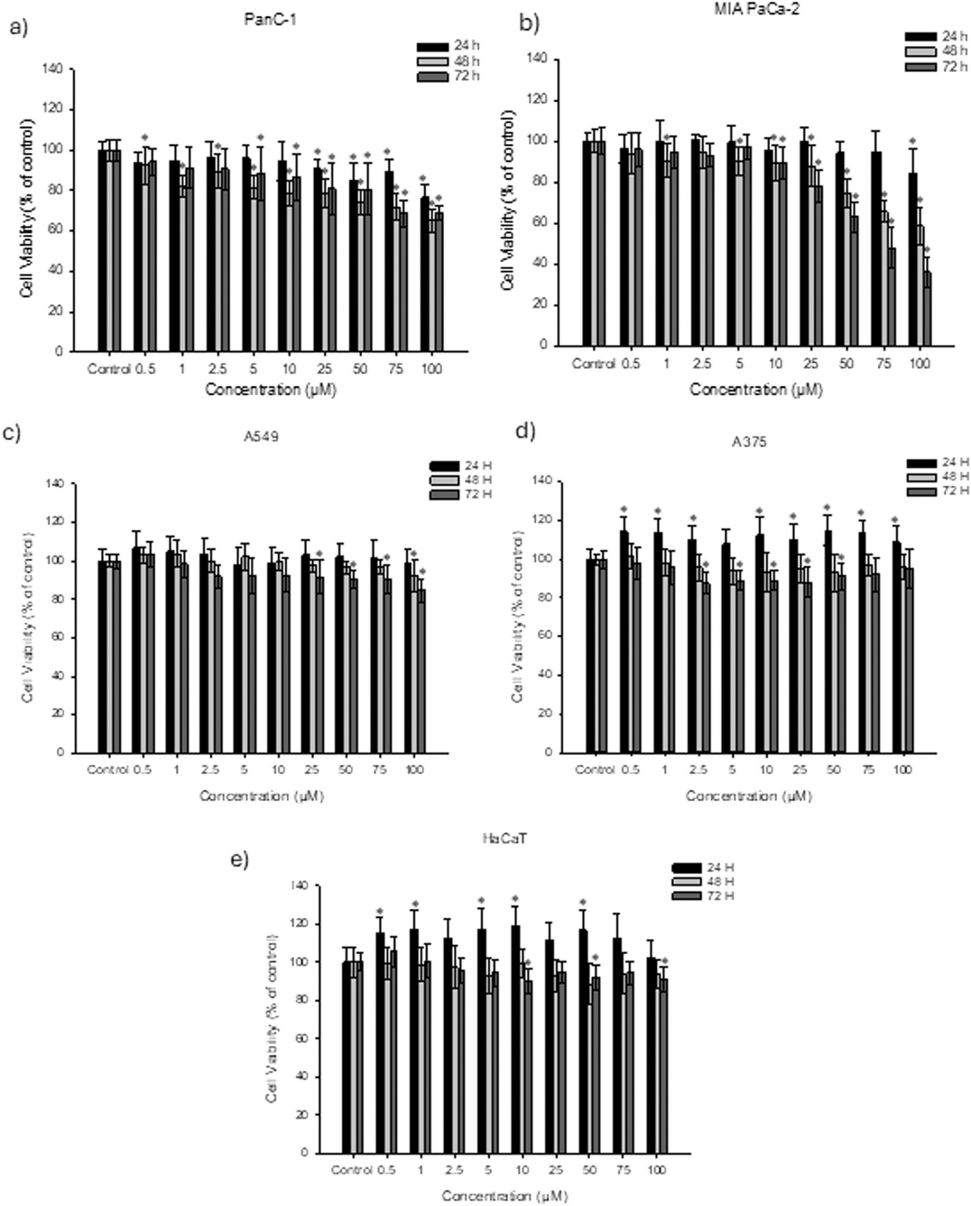
MTT assay to assess the **AcridPyMe** effect on the cell viability of a) PanC-1, b) MIA PaCa-2, c) A549, d) A375, and e) HaCaT cell lines. Data is represented by mean values ± standard deviation of three independent experiments with three technical replicates each. “*” is indicative of statistical significance in comparison to control (*p* < 0.05).

The cytotoxic effect of **AcridPy** and **AcridPyMe** on the cell viability of the two pancreatic tumor PanC-1 and MIA PaCa-2 cell lines, of the lung tumor A549 cell line, of the melanoma A375 cell line, and of the immortalized human keratinocyte HaCaT cell line was evaluated by MTT assay after 24, 48 and 72 h of exposure to each ligand (Fig. 9 and Fig. S23†).

As can be seen in Fig. 9 and Tables S4 and S5,† the cell viability of both pancreatic cancer cell lines seems to be inversely proportional to the concentration of the ligand, with the 72 h of exposure showing the most promising results. In contrast, **AcridPyMe** had none to little effect on the cell viability of the A549 (Fig. 9c, Table S6†), A375 (Fig. 9d, Table S7†) and HaCaT (Fig. 9e, Table S8†) cells, with viability values above 84%. In the PanC-1 cell line (Fig. 9a), cell viability decreased with statistical significance when exposed to **AcridPyMe** concentrations equal or superior to 25 μM for 24 h. After 72 h of exposure, a subtle cytotoxic effect can be seen at a concentration higher than 5 μM.

Regarding the MIA PaCa-2 cells (Fig. 9b), after 24 h of exposure, only the highest concentration of 100 μM showed reduced cell viability with statistical significance. After 72 h of exposure, only concentrations equal or above 10 μM affected MIA PaCa-2 cell viability. Comparing all cell lines, **AcridPyMe** showed a greater cytotoxic effect on the MIA PaCa-2 cell line above 50 μM, with a calculated IC_50_ of 72.69 μM after 72 h exposure. This value was not calculated for the other four cell lines since for the range of concentrations tested the decrease in viability of these cell lines did not reach 50%.

**AcridPy** showed a higher extent of cellular damage, when compared to **AcridPyMe**, decreasing cell viability of A549, HaCaT and A375 by about 50%, 60% and 30%, respectively, and by about 70% in the case of PanC-1 and MIA PaCa-2, after 72 h exposure (see Fig. S23 and Tables S9–S13†). However, these results were not considered for further *in vitro* experiments due to the evidence of formation of crystal-like aggregates in cellular medium (Fig. S23f†) in concentrations higher than 10 μM. To better understand their structure and formation process, the observed aggregates will be further studied.

#### 2.5.2. Fluorescence lifetime imaging microscopy

Fluorescence lifetime imaging microscopy (FLIM) is a technique highly sensible to the molecular environment that exploits the lifetime property of fluorescence and can be used in auto-fluorescent molecular imaging to study cellular metabolism. This microscopic approach is also very useful to study live animals, providing unique insights into cellular health in a non-destructive manner.^54^ By utilizing exogenous fluorophores, such as **AcridPyMe**, FLIM can track a variety of cellular and tissue activities, such as the course of disease and the effectiveness of drug efficacy.

**Fig. 10:**
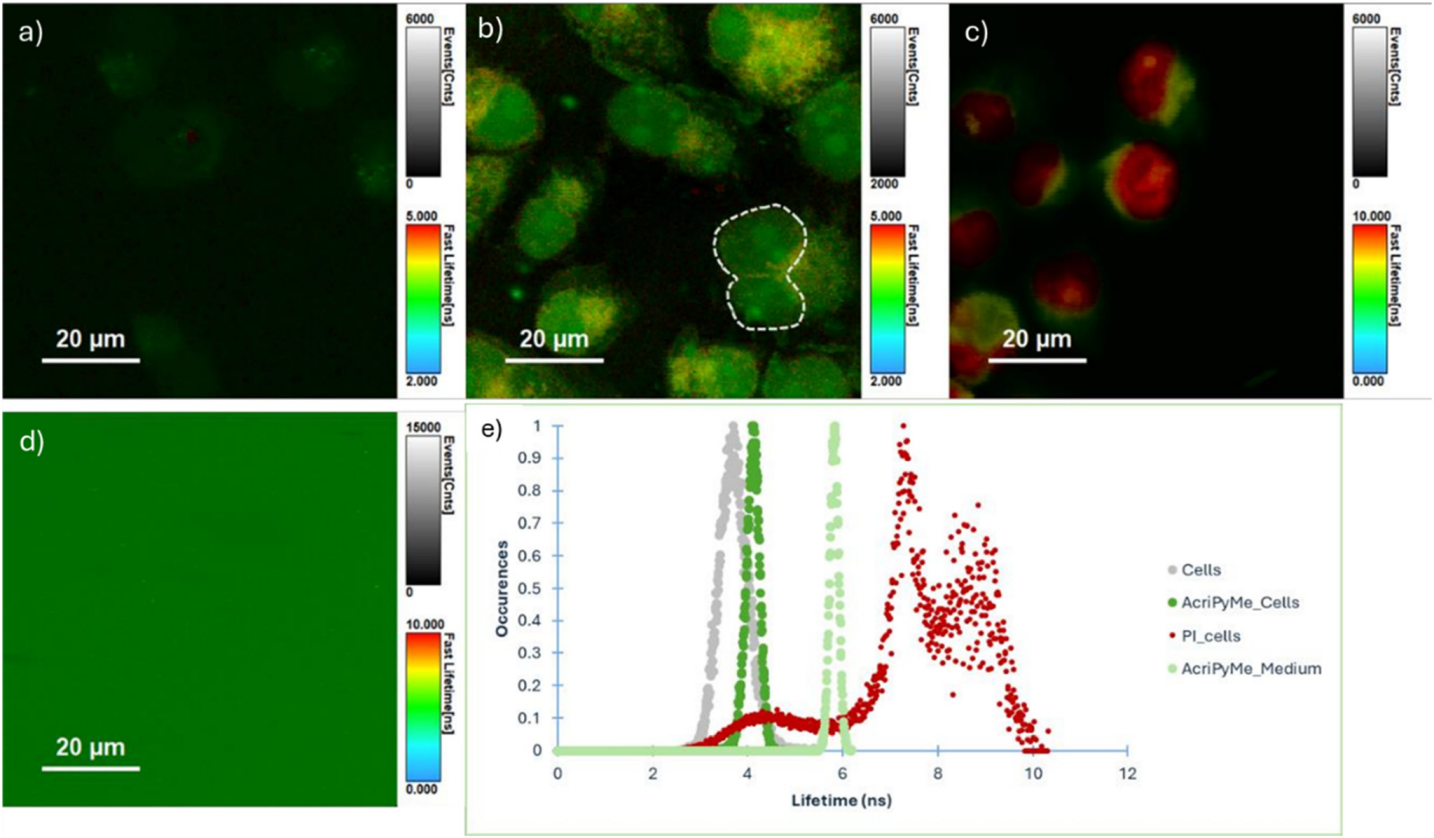
Fluorescence lifetime imaging microscopy of a) single cells; b) cells incubated with **AcridPyMe**; c) cells incubated with PI; d) **AcridPyMe** in incubation media; e) fluorescence lifetime histograms obtained for 1-D images.

In order to get time-resolved information about **AcridPyMe** spatial distribution in the cellular microenvironment, fluorescence lifetime images of MIA PaCa-2 cells incubated with **AcridPyMe** were acquired (Fig. 10b) and compared to fluorescence lifetime images obtained for single cells (Fig. 10a) and to cells incubated with propidium iodide (Fig. 10c), a well-known red fluorescent nuclear and chromosome counterstain.^55^ Fluorescence lifetime images obtained under similar experimental conditions (fluence rate and *λ*_exc_), as well as the histograms of average fluorescence lifetimes of each image are shown in Fig. 10a–c and e, respectively.

FLIM images of single cells show only residual fluorescence (*τ*_m_ = 3.7 ns) due to intrinsic fluorescence of endogenous fluorophores.^56^ After **AcridPyMe** incubation a significant increase in the fluorescence intensity of the cell occurs, as expected for the interaction with the fluorescent **AcridPyMe**. Simultaneously, an increase in the average fluorescence lifetime is observed (*τ*_m_ = 4.2 ns). The average fluorescence lifetimes of **AcridPyMe** in cells are slightly lower than the ones obtained in the ensemble measurements of an aqueous medium solution (*τ*_m_ = 5.8 ns), which agrees well with changes in the fluorophore's microenvironment after incubation. Although the spatial distribution of **AcridPyMe***τ*_m_ correlates with distinct cell regions (cytoplasm and nucleus), it seems to be preferentially located in the area that overlaps the nucleus region (green spots, Fig. 10b) as compared with the cell region marked with PI (Fig. 10c, red spots, *τ*_m_ = 8 ns). Interestingly, a dividing cell can be observed in Fig. 10b with the two nuclear regions marked with green spots (*τ*_m_ = 4.2 ns, area marked with a white dashed line).

#### 2.5.3. Cell cycle analysis

**Fig. 11:**
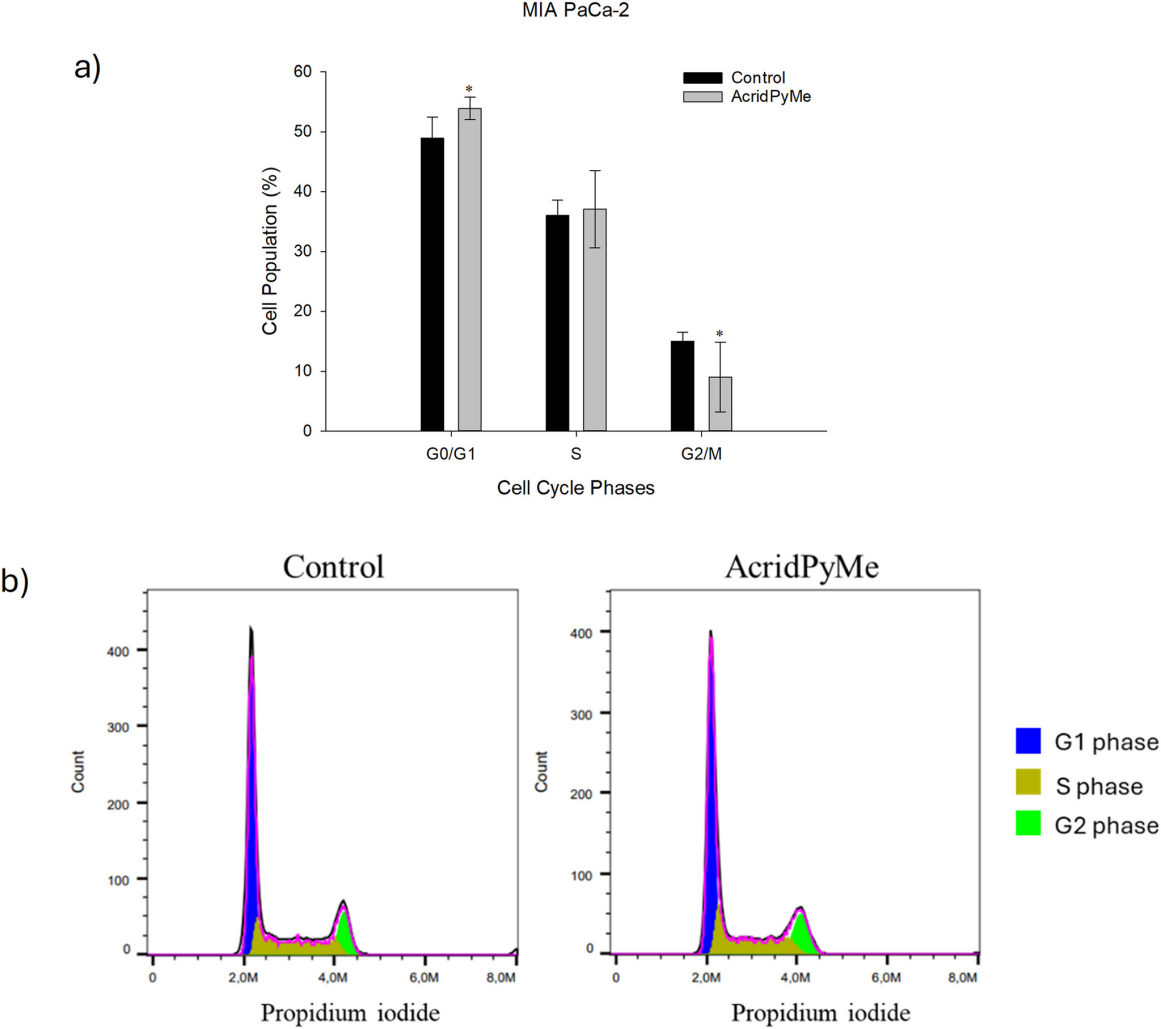
Cytotoxic effects of **AcridPyMe** on cell cycle distribution of MIA PaCa-2 cells. a) Cell cycle distribution (%); b) representative histograms of cell cycle analysis (arbitrary units). Data is represented by mean values ± standard deviation of two independent experiments with three technical replicates each and each replicate with at least 5000 events. “*” is indicative of statistical significance in comparison to control (*p* < 0.05).

To assess if the chemotherapeutic effect of **AcridPyMe** was associated with any interference within cell cycle progression, MIA PaCa-2 cells were exposed to the ligand for 72 h at a concentration corresponding to IC_50_ and the cell cycle was analyzed by flow cytometry. The cell cycle, a fundamental process for cell proliferation, is regulated by specific molecular mechanisms that ensure the integrity of the genome called cell cycle checkpoints. When DNA damage is identified, these checkpoints lead to cell cycle arrest, providing time for DNA repair or triggering cell death, depending on the nature of the damage.^57^ As seen in Fig. 11 and Table S14,† the percentage of MIA PaCa-2 cells at the G0/G1 phase was significantly increased by **AcridPyMe** from 49% (control) to 54% after 72 h, indicating a cell cycle arrest at the G0/G1 phase with a concomitant decrease in the percentage of cells at the G2/M phase.

#### 2.5.4. Cell apoptosis assay

**Fig. 12:**
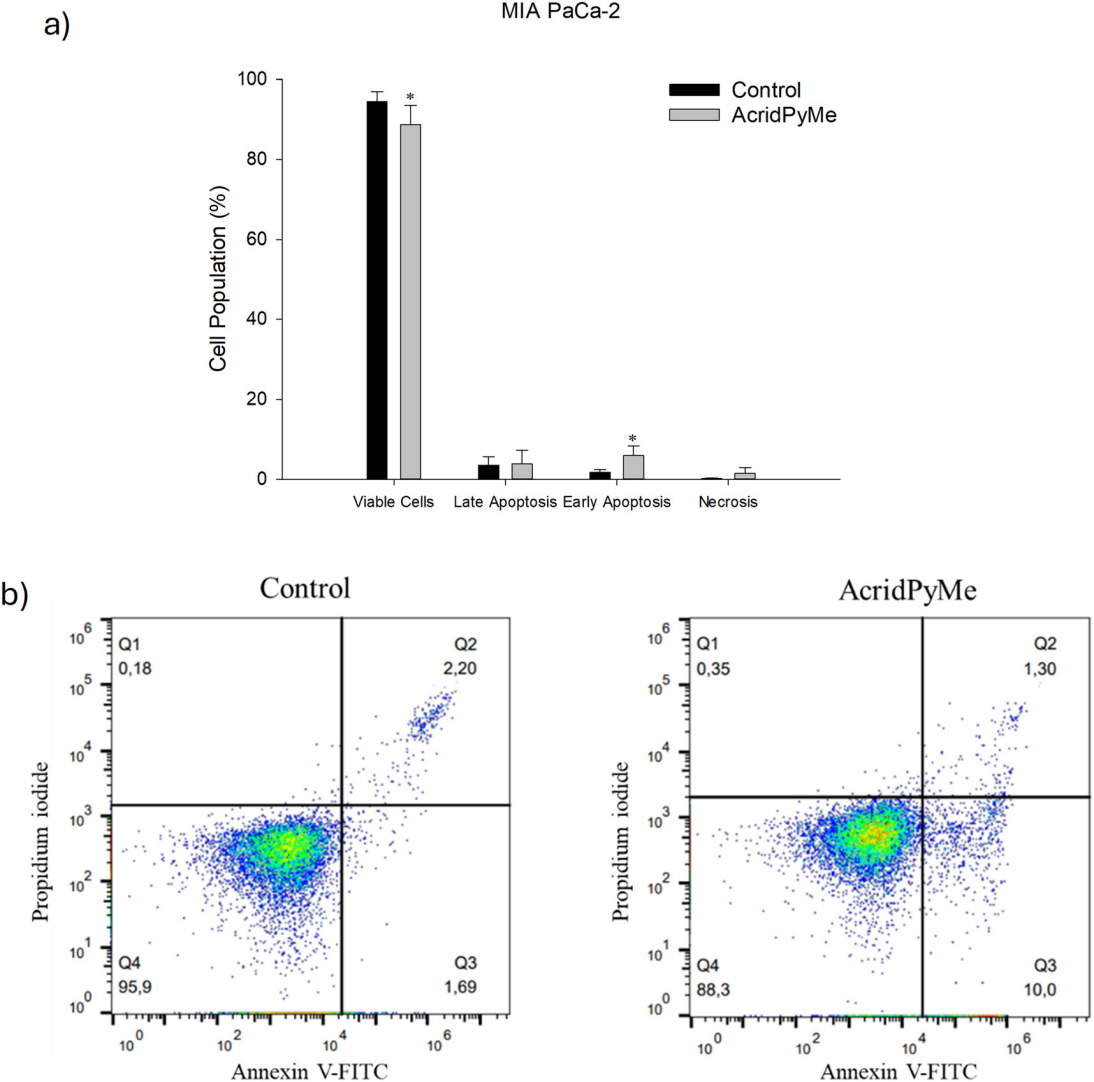
Cytotoxic effects of **AcridPyMe** on apoptosis in MIA PaCa-2 cells. a) Apoptotic cells (%) after treatment in groups analogous to viable and non-apoptotic; early and late apoptotic cells; b) representative histograms of Annexin V-FITC/propidium iodide. Data is represented by mean values ± standard deviation of two independent experiments with three technical replicates each and each replicate with at least 5000 events. “*” is indicative of statistical significance in comparison to control (*p* < 0.05).

Apoptotic cell death is characterized by various physiological changes, including the externalization of phosphatidylserine (PS) on the cell surface. In healthy cells, PS is confined to the inner leaflet of the plasma membrane.^58^ However, during the early stages of apoptosis, while cell membrane integrity remains intact, the asymmetry of the phospholipid membrane is lost, leading to the translocation of PS to the outer leaflet. This change can be detected using fluorescently labelled Annexin V conjugates. Annexin V is an ideal probe for PS because of its strong calcium-dependent binding affinity and specificity for the lipid, making it a reliable marker for early apoptosis.^59^ Therefore, the effect of treatment with **AcridPyMe** at IC_50_ for 72 h on cell apoptosis was also measured. As shown in Fig. 12 and Table S15,† there was a slight decrease in viable cells when treated with **AcridPyMe**, and an increase in cells in early apoptosis when treated with this ligand (∼10%), compared to the control. This treatment did not show any significant difference between the control and the samples in late apoptosis and necrosis.

## 3. Materials and methods

### 3.1. Chemicals

All commercial chemicals, solvents and reagents were acquired and used without any further purification. The DNA oligonucleotides were purchased from Eurogentec (Belgium). The DNA stock solutions (1 mM) were prepared in MilliQ waster, using the provided values for the molar extinction coefficient, and working solutions with desired concentrations were prepared with adequate buffer solutions. Stock solutions of **AcridPy** and **AcridPyMe** were prepared in dimethyl sulfoxide (DMSO) and stored at room temperature.

Thin layer chromatography (TLC) was carried out on precoated sheets with silica gel (Macherey-Nagel UV254). Column chromatography was carried out using silica gel (Merck, 35–70 mesh). ^1^H and ^13^C NMR spectra were recorded on a Bruker Avance 500 [500.16 MHz (^1^H), 125.77 MHz (^13^C)]. Deuterated methanol (CD_3_OD) was used as a solvent, and the internal reference was tetramethylsilane (TMS). The chemical shifts of the NMR spectra are expressed in *δ* (ppm) and the coupling constants (*J*) in Hz.

ESI-MS spectra were obtained using a linear ion trap mass spectrometer LXQ (ThermoFinnigan, San Jose, CA). Data were acquired, in positive mode, using a source voltage of 5 kV, capillary temperature of 275 °C and a sheath gas flow of 25 U. The Xcalibur data system (version 2.0, ThermoFinnigan, San Jose, CA) was used for data analysis.

High-resolution mass spectra (HRMS) were obtained, in positive mode, in a Q-Exactive® hybrid quadrupole Orbitrap® mass spectrometer (Thermo Fisher Scientific, Bremen, Germany). Data were acquired using 3.0 kV as spray voltage and interfaced with a HESI II ion source, capillary temperature of 320 °C and S-lens rf level 50. ESI analysis was performed at a flow rate of 10 μL min^−1^, nitrogen was used as sheath and auxiliary gas at flow rates of 5 and 1 (arb. units), respectively. Spectra were analyzed using the Xcalibur version 4.0 (Thermo Scientific, San Jose, CA, USA).

The melting point was obtained using a Büchi Melting Point B-540 within a range of 100 °C to 295 °C, with a gradient of 5 °C min^−1^. All the compounds are >95% pure by HPLC.

### 3.2. Preparation of DNA structures

A phosphate buffered saline (PBS) solution containing 20 mM phosphate buffer (10 mL of KH_2_PO_4_ 1 M and 200 μL of K_2_HPO_4_ 1 M) and 100 mM KCl was prepared with an adjusted pH of 6.8. Similarly, tris(hydroxymethyl)aminomethane (Tris)-NaCl buffer was prepared from a solution of 10 mL of Tris-HCl 100 mM and 10 mL of NaCl 1 M or LiCl 1 M.

The oligonucleotide solutions were heated at 90 °C for 10 minutes and left in ice for cooling for 15 minutes. All solutions were stored at −20 °C. The studied oligonucleotide sequences and corresponding abbreviations are presented in Table 1.

### 3.3. Synthesis of **AcridPy** and **AcridPyMe**

#### 3.3.1. Synthesis of **AcridPy**

To a mixture of (*E*)-3-iodo-2-(4-methoxystyryl)-1-methylquinolin-4(1*H*)-one (50 mg, 0.120 mmol), 0.1 equiv. of Pd(OAc)_2_ (0.012 mmol, 2.69 mg), 1.0 equiv. of K_2_CO_3_ (0.120 mmol, 16.56 mg), 0.1 equiv. of TBAB (0.012 mmol, 3.90 mg) and dried DMF (4 mL) were added 2.5 equiv. of 4-vinylpyridine (0.300 mmol, 32.3 μL). The mixture was then heated to 115 °C and stirred for 24 h under nitrogen atmosphere. DMF was then evaporated. The remaining solid was dissolved in ethyl acetate and washed with water. The collected organic layer was dried over anhydrous sodium sulfate and concentrated and the product was then isolated by preparative TLC, using a mixture of dichloromethane/methanol (9.5 : 0.5) as eluent. The compound **AcridPy** was obtained with a yield of 52%. A molar extinction coefficient (*ε*_306_) of 22 637 M^−1^ cm^−1^, in PBS, was found (Fig. S7†). The purity of **AcridPy** was determined through HPLC/UV-vis analysis, obtaining a purity higher than 99% (Fig. S8†).

3-(4-Methoxyphenyl)-10-methyl-2-(pyridin-4-yl)acridin-9(10*H*)-one (**AcridPy**), brownish solid (yield 52%, 24.3 mg), mp: >235.0 °C (decomposition); ^1^H NMR (500.16 MHz, CD_3_OD), *δ*_H_ = 8.56 (s, 1H, H-1), 8.54 (d, *J* = 5.8 Hz, 2H, H-3′,5′), 8.50 (dd, *J* = 7.6, 1.6 Hz, 1H, H-8), 7.92–7.86 (m, 2H, H-6, H-5), 7.82 (br s, 1H, H-4), 7.55 (d, *J* = 5.8 Hz, 2H, H-2′,6′), 7.43 (t, *J* = 7.6 Hz, 1H, H-7), 7.26 (d, *J* = 8.6 Hz, 2H, H-2′′,6′′), 6.95 (d, *J* = 8.6 Hz, 2H, H-3′′,5′′), 4.04 (s, 3H, –NCH_3_), 3.84 (s, 3H, 4′-OCH_3_) ppm. ^13^C NMR (CD_3_OD, 125.77 MHz), *δ*_C_ = 178.0 (C-9), 160.0 (C-4′′), 154.0 (C-2), 146.5 (C-3), 145.0 (C-1), 143.1 (C-4b), 142.9 (C-4a), 134.6 (C-6), 131.6 (C-1′′), 131.0 (C-2′′,6′′), 129.8 (C-1′), 129.3 (C-2′,6′), 126.6 (C-8), 126.1 (C-3′,5′), 122.1 (C-8a), 121.9 (C-7), 120.5 (C-9a), 117.9 (C-4), 115.7 (C-5), 113.7 (C-3′′,5′′), 54.4 (*N*CH_3_), 33.2 (4′′′-OCH_3_) ppm. MS (ESI) *m*/*z*: 393.3 [M + H]^+^. HRMS-ESI(+): *m*/*z* calcd. for C_26_H_20_N_2_O_2_ 393.1598 [M + H]^+^, found 393.1595.

#### 3.3.2. Methylation of **AcridPy**

To a solution of **AcridPy** (0.064 mmol, 25 mg) in DMF (4 mL) were added 15 equiv. of iodomethane (0.956 mmol, 60 μL). This mixture was stirred and kept at 40 °C for 24 h. After, diethyl ether was added to form a precipitate, which was filtered and rinsed with additional diethyl ether and was left to dry under vacuum. The compound **AcridPyMe** was obtained with a yield of 67%. A molar extinction coefficient (ε_320_) of 5348 M^−1^.cm^−1^, in PBS, was found (Fig. S7†). The purity of **AcridPyMe** was also determined through HPLC/UV-vis analysis, obtaining a purity higher than 99% (Fig. S9†).

4-[3-(4-Methoxyphenyl)-10-methyl-9-oxo-9,10-dihydroacridin-2-yl]-1-methylpyridin-1-ium, yellow solid (yield 67%, 17.41 mg), mp: >258.0 °C (decomposition); ^1^H NMR (500.16 MHz, CD_3_OD), *δ*_H_ = 8.73 (s, 1H, H-1), 8.72 (d, *J* = 6.8 Hz, 2H, H-3′,5′), 8.52 (dt, *J* = 8.0, 1.2 Hz, 1H, H-8), 7.95–7.93 (m, 2H, H-5, H-6), 7.91 (br s, 1H, H-4), 7.88 (d, *J* = 6.8 Hz, 2H, H-2′,6′), 7.47 (ddd, *J* = 8.0, 4.7, 3.2 Hz, 1H, H-7), 7.32 (d, *J* = 8.7 Hz, 2H, H-2′′,6′′), 7.00 (d, *J* = 8.7 Hz, 2H, H-3′′,5′′), 4.36 (s, 3H, *N*CH_3_-Py), 4.12 (s, 3H, *N*CH_3_), 3.85 (s, 3H, 4′′-OCH_3_) ppm. ^13^C NMR (CD_3_OD, 125.77 MHz), *δ*_C_ = 178.0 (C-9), 160.1 (C-4′′), 157.7 (C-2), 146.4 (C-3), 144.4 (C-1), 143.9 (C-1′), 143.9 (C-4a), 143.0 (C-4b), 134.8 (C-6), 131.0 (C-2′′,6′′), 130.8 (C-1′′), 130.2 (C-3′,5′), 127.7 (C-2′,6′), 126.7 (C-8), 122.3 (C-9a), 122.3 (C-7), 120.7 (C-8a), 118.5 (C-4), 116.0 (C-5), 114.1 (C-3′′,5′′), 54.6 (4′′-OCH_3_), 46.65 (NCH_3_-Py), 33.7 (NCH_3_) ppm. MS (ESI) *m*/*z*: 407.3 M^+^. HRMS-ESI(+): *m*/*z* calcd. for C_27_H_23_N_2_O_2_ 407.1760 M^+^, found 407.1747.

### 3.4. UV–vis spectroscopy

The UV–vis absorption spectra were recorded on a Shimadzu UV-2501-PC spectrophotometer. All measurements were restricted to a range from 250 to 550 nm, using a quartz cuvette of 1 cm length, at room temperature. Successive additions of **AcridPy** or **AcridPyMe** were made in PBS to determine the extinction coefficients (*ε*) of these molecules by a calibration curve, using the Beer–Lambert Law

1 *A* = *ε* × *b* × *c*

where *A* is the absorbance of the molecule, *b* is the optical path of the laser beam and *c* is the concentration of the ligand.

### 3.5. Fluorescence studies

#### 3.5.1. Fluorescence quantum yield determination

For the determination of the fluorescence quantum yield (*Φ*) of both ligands, absorbance and fluorescence spectra were recorded on a Shimadzu UV-2501-PC spectrophotometer and a Horiba FluoroMax-4 spectrofluorometer, respectively. In this work we used a relative method for the determination of *Φ*s, based on the equation2
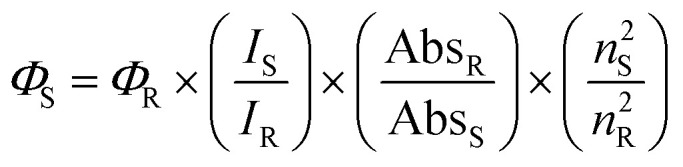
where *Φ*s is the fluorescence quantum yield, *I* is the integrated fluorescence intensity, Abs is the absorbance, *n* is the refractive index of the solvent and the subscript S refers to the sample, while the subscript R refers to the reference fluorophore, being fluorescein (*Φ*_R_ = 0.79, ethanol) and anthranilic acid (*Φ*_R_ = 0.60, ethanol) the ones used in this experiment.^60,61^

Rearranging the previous equation, we can determine the *Φ* of our sample by measuring the absorbance and fluorescence intensity of several standard solutions (in DMSO) with known concentration and by plotting the integrated fluorescence intensity as a function of absorbance (Fig. S10†), resulting in3
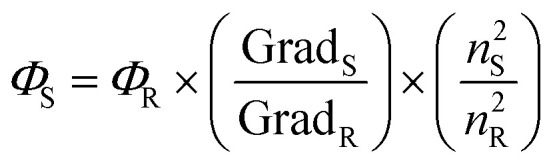
where Grad_S_ is the slope of the calibration curve of the known fluorophore and Grad_R_ is the slope of the curve of our sample.

#### 3.5.2. Fluorescence titrations

The fluorescence spectra were recorded on a Horiba FluoroMax-4 spectrofluorometer at room temperature with a quartz cuvette of 1 cm length. Ligands were excited at 306 and 320 nm for **AcridPy** and **AcridPyMe**, respectively, the emission spectra being recorded between 315 and 590 nm for **AcridPy** with excitation and emission slits fixed at 3 nm, and between 330 and 630 nm for **AcridPyMe** with both slits fixed at 4 nm.

Titrations of ligand solutions at 10 μM with the appropriate oligonucleotide at concentrations ranging from 0 to 16.7 μM were performed in the same equipment. The apparent dissociation constants (*K*_D_) were obtained from the changes observed in fluorescence spectra of each oligonucleotide–ligand interaction. The obtained data was converted into the fraction of ligand that is bound to the DNA (*α*), and plotted using the expression:4
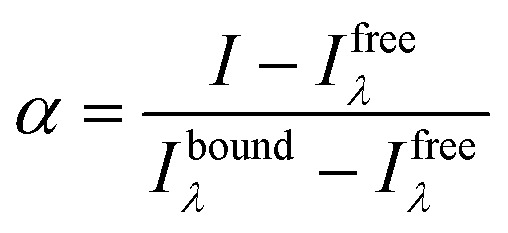
where *I* represents the fluorescence intensity of each ratio of ligand/G4, with *I*^free^ and *I*^bound^ being the fluorescence intensity of the free and bound ligand, respectively. These points were then fitted into a hyperbolic/sigmoidal Hill function and *K*_D_ was determined for each ligand–oligonucleotide interaction.

Each *K*_D_ was then determined following the saturation binding model, using *OriginPro* 2016, described by the following equation:5
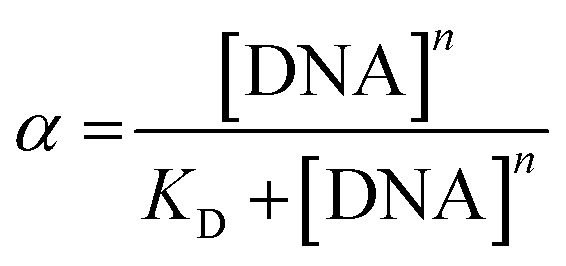
where *α* is the fraction of the bound ligand, [DNA] is the concentration of DNA and *n* is the Hill constant, which describes the cooperativity of the binding between the ligand and the DNA.

#### 3.5.3. Fluorescence intercalator displacement assay

Fluorescence Intercalator Displacement (FID) assay was performed for the most promising DNA–ligand pairs identified from the previous data obtained from the fluorescence titrations using a stock solution of thiazole orange (TO) at 35 μM and the corresponding oligonucleotide at a concentration of 10 μM. These solutions were mixed in a 1 : 1 proportion and shaken at 500 rpm for 10 min. The fluorescence was then measured in a Horiba FluoroMax-4 spectrofluorometer, with an excitation wavelength of 485 nm in the emission range of 510–750 nm and slits at 10 nm.

Similarly, FID was also performed using Hoechst 33258 (3.5 μM) and maintaining the other conditions mentioned above. In this case, the spectra were recorded at an excitation wavelength of 340 nm and an emission range between 350 and 600 nm with both excitation and emission slits set at 3 nm.

### 3.6. Circular dichroism spectroscopy

#### 3.6.1. CD spectra

Circular dichroism (CD) sample spectra were registered in a Jasco J-1500 spectrometer. All measurements were made in a quartz cell of 1 cm at 20 °C. The CD spectrum was recorded with a scanning speed of 100 nm min^−1^ with a data pitch of 0.5 nm, within a range of 220 to 320 nm. Bandwidth was set to 1 nm with a Digital Integration Time (DIT) set to 4 sec.

For CD titration, solutions of oligonucleotides (4 μM) were prepared in 20 mM phosphate buffer (10 mM KH_2_PO_4_, 10 mM K_2_HPO_4_, pH 7.1) containing 100 mM KCl for c-MYC and G4 Tel, and 100 mM NaCl also for G4 Tel Na^+^. The ligands were added to the quartz cell solution within a range of 0 to 24 μM.

#### 3.6.2. CD melting experiments

CD melting was performed in a temperature range of 20 to 100 °C with a heating rate of 2 °C min^−1^ and monitoring the ellipticity at 265, 290 and 295 nm, depending on the selected oligonucleotide (MYC or G4 Tel), and with a bandwidth of 1 nm and DIT of 2 sec. Spectra were recorded at 0 and 16 μM for both ligands. All data were converted into fraction folded (*θ*) using the following equation:6
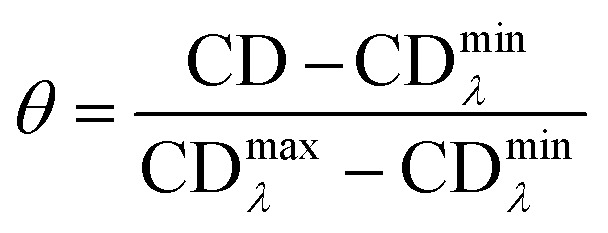
The *θ* values were then fitted into a Boltzmann distribution, using *OriginPro* 2016, aiming to obtain the melting temperature *T*_m_.

### 3.7. *In silico* studies

#### 3.7.1. Compound force field derivation

The 2D structures of **AcridPy** and **AcridPyMe** were generated using the 2D sketcher tool in Maestro. The 3D conformers were generated using LigPrep in Maestro with default settings (Schrodinger 2022-3 package). An in-house protocol was used to generate the force field for both compounds using the standard AMBER protocol.^62,63^ First, the molecular electrostatic potential (MEP) for **AcridPy** and **AcridPyMe** was calculated *via* Gaussian 09 at the HF/6-31* level. Second, the geometry of the compounds was performed at the same level. Third, MEP under the restrained electrostatic potential (RESP) method with two-stage fitting was used to calculate the atomic partial charges for both ligands. The MOL2 files of **AcridPy** and **AcridPyMe** were generated in Table S3.†

#### 3.7.2. Simulation system protocols

The structure models of the Pu22 MYC G-quadruplex (PDB ID: 1XAV),^64^ KRAS G-quadruplex (PDB ID: 5I2V),^65^ human telomeric repeat G-quadruplex (PDB ID: 143D)^62^ and ds26 were obtained and/or constructed as per our previous protocol. The ligands **AcridPy** and **AcridPyMe** were displaced 10 Å away from each nucleic acid structure for sufficient sampling during the simulation. The simulation systems were prepared *via* 1) neutralizing the negative DNA charge with sufficient K^+^ cations; 2) establishing a 0.1 M KCl salt concentration; 3) solvating the system in an OPC water box of truncated octahedron with a water buffer distance of 10 Å from the DNA structure. The following force fields were applied to the DNA structure (OL15, which contains several corrections of torsion angle parameters (parm99bsc0 + vOL4 + e/fOL1 + bOL1)^66–69^), water molecules (OPC), K^+^ ions within the G4 DNA structures (Cheatham K+^70,71^) and all other parameters (GAFF2).

#### 3.7.3. MD simulation protocols

Each simulation system was run using the AMBER22 molecular dynamics (MD) simulation package. Our protocol is very similar to what we previously described.^72–74^ We first randomize the position of all ions and waters using a pre-run of 1000 ps at 500 K before performing a production run at 300 K. A total of sixteen independent production runs (trajectories of 500 ns each) at 300 K with random initial velocities were performed, two for each of the eight systems. The following describes the detailed simulation protocol for a single production run. The system density was equilibrated by performing a 1.0 ns MD simulation at NPT (constant temperature and pressure) and production dynamics at NVT (constant volume and temperature). Then, to constrain all hydrogen-containing bonds and enable a 2.0 fs time step in the simulations, the SHAKE algorithm was used.^75^ Next, long-range electrostatic interactions were treated *via* the Particle-mesh Ewald (PEM) method (with settings set for a charge grid spacing of 1.0 Å, fourth-order B-spline charge interpolation and direct sum tolerance of 10–5) under periodic boundary conditions.^76^ Short-range non-bonded interactions were treated using a cut-off distance of 10 Å, whereas long-range van der Waals interactions were treated using a uniform density approximation. Nonbonded forces were calculated using a two-stage RESPA approach to reduce computation time. When decomposed, short-range forces and long-range forces were calculated at every one and two timesteps, respectively.^77^ The temperature was controlled using the Langevin thermostat and a coupling constant of 2.0 ps. All trajectories were saved every 50.0 ps for post-simulation analysis.

#### 3.7.4. Simulation convergence

Receptor root mean-squared deviation (R-RMSD) was calculated for the heavy atoms of the nucleic acid sugar phosphate backbone and ligand root mean-squared deviation (L-RMSD) was calculated for all heavy atoms in the **AcridPy** and **AcridPyMe** ligands for each timestep compared to their starting structures (0 ns). The trajectory is suggested to reach a convergence point when the R-RMSD and L-RMSD values do not significantly change, indicating that chemical equilibrium can be approximated. At this equilibrium, major conformations of both solute and ligand binding conformations can be observed. RMSD graphs and values for **AcridPy** and **AcridPyMe** systems are presented in the main text and ESI.†

#### 3.7.5. Calculating atom contacts between **AcridPy** and **AcridPyMe** to each nucleic acid

The total atom contacts formed between each ligand and the nucleic acid structure surface over the course of the trajectory were analysed using an atom distance cutoff of 3.0 Å to determine the preference of **AcridPy**/**AcridPyMe** to each nucleic acid structure.

#### 3.7.6. Ligand clustering analysis

The positions of the C1 atom for **AcridPy** and **AcridPyMe** for each ns of simulation time were mapped to each nucleic acid structure to characterize ligand binding cluster(s) and to differentiate the binding modes between each compound. The structures have been generated in the main text.

#### 3.7.7. Last snapshot generation

The last snapshot structures obtained from the last timestep of simulation time of **AcridPy** and **AcridPyMe** to each nucleic acid structure were generated and placed in the ESI.†

#### 3.7.8. Structure visualization

VMD 1.9.3. was used to generate all structure representations.

### 3.8. Biological assays

#### 3.8.1. Cell culture

In this work, two pancreatic tumor cell lines were used: PanC-1, pancreas adenocarcinoma cells, and MIA PaCa-2, undifferentiated human pancreatic carcinoma, both kindly provided by Dr. Sónia Melo (i3s, Porto, Portugal). The immortalized human keratinocyte HaCaT cells were obtained from Cell Lines Services (Eppelheim, Germany). The A549, human non-small cell lung cancer, and A375, human malignant melanoma, cell lines were acquired from the European Collection of Authenticated Cell Cultures (ECACC). All cell lines were individually cultured in high-glucose DMEM (PAN-Biotech, Aidenbach, Germany), which was supplemented with 10% fetal bovine serum (FBS, PAN-Biotech, Aidenbach, Germany), 2 mM l-glutamine and 1% penicillin/streptomycin (100 U mL^−1^ penicillin, 100 μg mL^−1^ streptomycin, Grisp, Porto, Portugal). The cell cultures were incubated at 37 °C with 5% CO_2_. Upon reaching 75–80% confluence, the cells were collected and further employed for cytotoxicity assays.

#### 3.8.2. Cell viability assays

The cytotoxic ability of **AcridPyMe** on PanC-1 and MIA PaCa-2, A549, A375 and HaCaT cells was evaluated by MTT assay [3-(4,5-dimethyl-2-thiazolyl)-2,5-diphenyl-2H-tetrazolium bromide], Sigma-Aldrich, St. Louis, Missouri, USA. Cells were exposed to **AcridPyMe** at concentrations 0.5 to 100 μM for 24, 48 and 72 h. After exposure, 50 μL of MTT (1.0 mg mL^−1^ in PBS, PAN-Biotech, Aidenbach, Germany) was added and the plates were incubated for another 3 h at 37 °C. Then, the medium with MTT was removed and 150 μL of dimethyl sulfoxide (DMSO, ≥99.5%, Sigma-Aldrich, St. Louis, Missouri, USA) was added to dissolve the produced formazan crystals. The plates were shaken in the dark for 1 h and then the absorbance was read in a microplate reader (Synergy HT® Multi-Mode, BioTek®, Vinooski, VT, USA) at 570 nm. Cells without exposure to drugs and incubated at 37 °C were used as control. The cell viability was calculated using the following equation:7
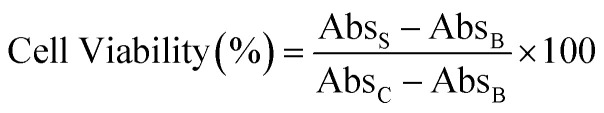
where Abs means the measured absorbance of the solution and the subscripts S, B and C stand for sample, blank and control, respectively.

#### 3.8.3. Confocal microscopy

MIA PaCa-2 cells were grown at a density of 1 × 10^4^ cells per mL on glass coverslips in 12 well plates, and then incubated for 72 h at 37 °C and 5% CO_2_ to facilitate cell attachment. After that, cells were exposed to 50 μM **AcridPy** for 72 h. Cells were then washed 3 times with warm PBS and fixed with 4% paraformaldehyde in PBS for 15 min at room temperature. After being washed with cold PBS, cells were washed with abundant Milli-Q water and the coverslips mounted onto glass slides with 4′,6-diamidino-2-phenylindole (DAPI)-containing Vectashield mounting medium (Vector Labs). Microphotographs were captured using a Zeiss LSM 880LSM 510 META confocal microscope (Zeiss, Jena, Germany) equipped with a Plan-Neofluor 63×/1.4 oil immersion objective. DAPI fluorescence was collected at 420–480 nm (*λ*_exc_ = 405 nm) and **AcridPy** fluorescence at 420–480 nm (*λ*_exc_ = 405 nm).

#### 3.8.4. Fluorescence lifetime imaging microscopy

MIA PaCa-2 cells were grown at a density of 1 × 10^4^ cells per mL on glass coverslips in 12 well plates and then incubated for 72 h at 37 °C and 5% CO_2_ to facilitate cell attachment. After that, cells were exposed to 50 μM **AcridPyMe** for 72 h. Cells were then washed 3 times with warm PBS and fixed with 4% paraformaldehyde in PBS for 15 min at room temperature. After being washed with cold PBS, cells were labelled with propidium iodide (PI, ≥94%, Merck KGaA, Darmstadt, Germany). After 20 min incubation, cells were washed with abundant Milli-Q water and the coverslips mounted onto glass slides with mounting medium (Vector Labs). FLIM measurements were performed with a time-resolved confocal microscope (MicroTime 200, PicoQuant GmbH). Briefly, the excitation at 405 nm was carried out by a pulsed diode laser at a repetition rate of 20 MHz, through a water immersion objective (UPLSAPO 60×, N.A. 1.2, Olympus). The fluorescence was detected with a single-photon counting avalanche diode (SPAD) (PerkinElmer) whose signal is processed by TimeHarp 200 TC-SPC PCboard (PicoQuant) working in the time-tagged time resolved (TTTR) operation mode. For point-by-point measurements, fluorescence decays were collected from random points chosen within the cell structure.

#### 3.8.5. Cell cycle analysis

MIA PaCa-2 cells were seeded in 12-well plates at a density of 1 × 10^4^ cells per mL. After adhesion, cells were exposed to **AcridPyMe** at the concentration equivalent to the estimated IC_50_ at 72 h of exposure (72.69 μM). Thereafter, cells were incubated at 37 °C for 72 h. Then, cells were washed with PBS, trypsinized and collected, and centrifuged at 700 × *g* for 5 min. After supernatant removal, the cell pellets were washed in PBS, and then fixed with 85% cold ethanol. Samples were stored at −20 °C until analysis.

Prior to analysis, samples were centrifuged at 700 *g* for 5 min, and ethanol was removed. Cells were resuspended with PBS, filtered with nylon filter membranes, and incubated with 50 μg mL^−1^ of RNase (Merck KGaA, Darmstadt, Germany) for 10 min. Next, 50 μg mL^−1^ of propidium iodide (PI, ≥94%, Merck KGaA, Darmstadt, Germany) was added and samples were incubated for at least 20 min in the dark. Cell cycle distributions were analyzed on an Attune® Acoustic Focusing Cytometer (Applied Biosystems, Thermo Fischer Scientific, Waltham, MA, USA) flow cytometer. Two independent assays with three replicates each were completed, and at least 5000 events were obtained. The percentage of cells in each cell cycle phase was determined using the FlowJo software (FlowJo LLC, Ashland, OR, USA).

#### 3.8.6. Cell apoptosis assay

Determination of apoptosis was performed with the Annexin V-FITC Apoptosis Detection Kit (BD Biosciences, Franklin Lakes, NJ, USA). MIA PaCa-2 cells were seeded in 6-well plates at a density of 1 × 10^4^ cells per mL, as previously described, and incubated with 72.69 μM AcridPyMe for 72 h. After treatment, cells were gently collected, counted, and washed twice in PBS after centrifugation (300 × *g*, 5 min, 4 °C). Cells were then resuspended in 1× binding buffer and Annexin V-FITC and PI were added to 100 μL of cell suspension (1 × 10^5^ cells), and incubated for 15 min. Data were acquired in the following hour using an Attune® Acoustic Focusing Cytometer (Applied Biosystems, Thermo Fischer Scientific, Waltham, MA, USA). Further analysis was performed with FlowJo software (FlowJo LLC, Ashland, OR, USA).

#### 3.8.7. Statistical analysis

Results are represented as the mean ± standard deviation. Data were analyzed with SigmaPlot version 14.0 (Systat Software, San Jose, CA, USA) for Windows. Data were analyzed by one-way ANOVA (*p* < 0.05) followed by Dunnett's test (*p* < 0.05).

## 4. Conclusions and future perspectives

The results presented in this work show that these novel acridone derivatives are promising G4 binding ligands, with the ability to stabilize these secondary DNA structures. Fluorescence experiments demonstrated their ability to bind and stabilize G4 DNA, with a particular affinity of **AcridPy** for telomeric G4 DNA and of **AcridPyMe** for MYC oncogene sequences. The cationic derivative **AcridPyMe** showed high selectivity towards G4 structures, rather than duplex DNA. CD melting assays have also shown an enhancement in the melting temperature of the ligand–G4 adduct, especially in the case of the **AcridPyMe**–MYC adduct, with a difference greater than 10 °C confirming the stabilization of MYC induced by the presence of the ligand. The results observed from *in silico* studies point to the occurrence of binding on top and/or groove binding modes of both ligands, which was supported by the fluorescent displacement assays performed earlier.

The biological *in vitro* assays also demonstrated a good cytotoxic effect of **AcridPyMe** in the MIA PaCa-2 cell line, which was sustained by cell viability, cell cycle and apoptosis assays, that showed cell cycle arrest at the G0/G1 phase, a decrease in viable cells and an increase in cells in early apoptosis when treated with **AcridPyMe**. Internalization showed that **AcridPyMe** appears to be distributed in the cellular cytoplasm and nucleus, apparently being preferentially located in the area that overlaps the nucleus region.

Overall, the results of this study led to the proposal of the new **AcridPyMe** ligand as a promising candidate to target the MYC oncogene, effectively stabilizing the signature G4 structures present in this type of DNA sequence and being able to compromise cell proliferation. These findings may contribute, in the future, to the development of G4-targeted cancer therapeutics.

## Funding

This work received financial support from PT national funds (FCT/MCTES, Fundação para a Ciência e Tecnologia and Ministério da Ciência, Tecnologia e Ensino Superior) through the project unit UID/50006/2020 (Laboratório Associado para a Química Verde - Tecnologias e Processos Limpos ) through national funds and UID Centro de Estudos do Ambiente e Mar (CESAM) + LA/P/0094/2020. CIV Ramos thanks FCT for funding through program DL 57/ 2016 (CDLCTTRI-047-88-ARH/2018). D. Salvador thanks FCT for the PhD grant (2022.11049.BD). V. V. Serra thanks FCT for the institutional funds provided to Centro de Química Estrutural (UIDB/00100/2020 and UIDP/00100/2020), to the Institute of Molecular Sciences (LA/P/0056/2020), and her research contract (IST/ID/113/2018) funded by national funds (OE), through FCT.

## Data availability

Data will be made available on request.

## Conflicts of interest

There is no conflict of interest to declare.

## Supplementary Material

MD-OLF-D4MD00959B-s001
