# Supplementary material for: New acridone derivatives to target telomerase and oncogenes – an anticancer approach
Source: RSC Med Chem. 2025 Apr 17;16(6):2785–807. doi: 10.1039/d4md00959b (PMC12004264; doi:10.1039/d4md00959b)
Supplement: MD-016-D4MD00959B-s001 [file MD-016-D4MD00959B-s001.pdf]

Supplementary Information (SI) for RSC Medicinal Chemistry.
This journal is © The Royal Society of Chemistry 2025

New Acridone Derivatives to Target Telomerase and Oncogenes – an Anticancer Approach

Supplementary Information

Tiago Marques[1], Diana Salvador[2,3], Helena Oliveira[2], Vanda V. Serra[4], Nicholas Paradis[5], Chun Wu[5], Vera Silva[1,*], Catarina I. V. Ramos[1,*]

[1] LAQV-REQUIMTE, Department of Chemistry, University of Aveiro, 3810-193 Aveiro, Portugal

[2] CESAM-Centre for Environmental and Marine Studies, Department of Biology, University of Aveiro, 3810-193 Aveiro, Portugal

[3] CICECO, Aveiro Institute of Materials, Department of Chemistry, University of Aveiro, 3810-193 Aveiro, Portugal

[4] Centro de Química Estrutural, Institute of Molecular Sciences, Instituto Superior Técnico, Universidade de Lisboa, Av. Rovisco Pais 1, 1049-001, Lisboa, Portugal

[5] Department of Chemistry and Biochemistry, Rowan University, Glassboro, New Jersey

1. Synthesis and characterization of **AcridPy** and **AcridPyMe**

1.1. Nuclear Magnetic Resonance Spectra

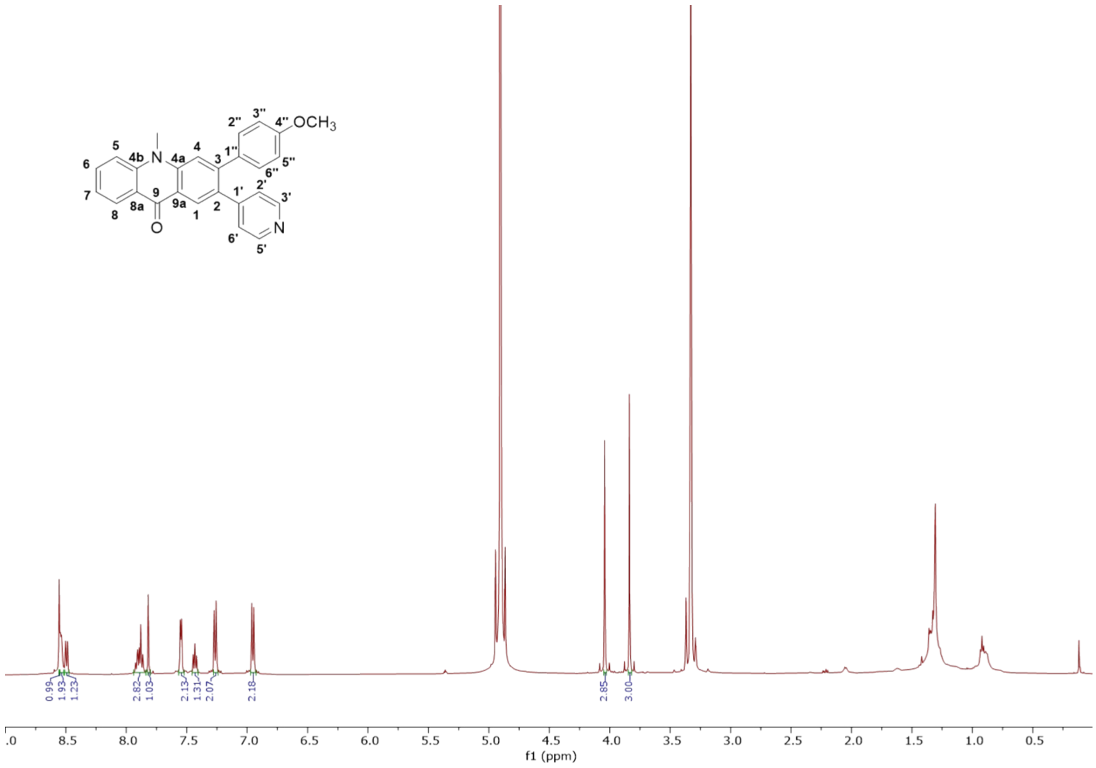

Figure S1. ^{1}H NMR spectrum of **AcridPy** (500.16 Hz, CD_3OD).

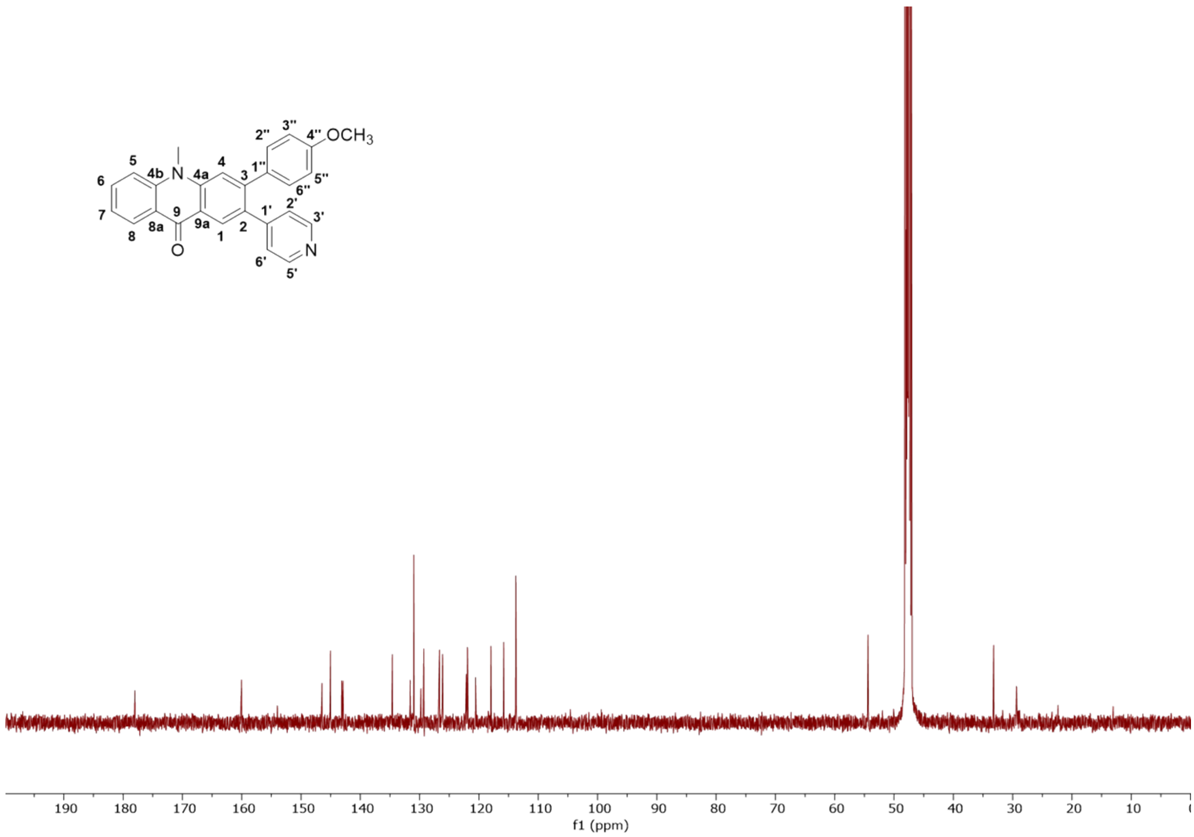

Figure S2. ^{13}C NMR spectrum of **AcridPy** (125.77 Hz, CD_3OD).

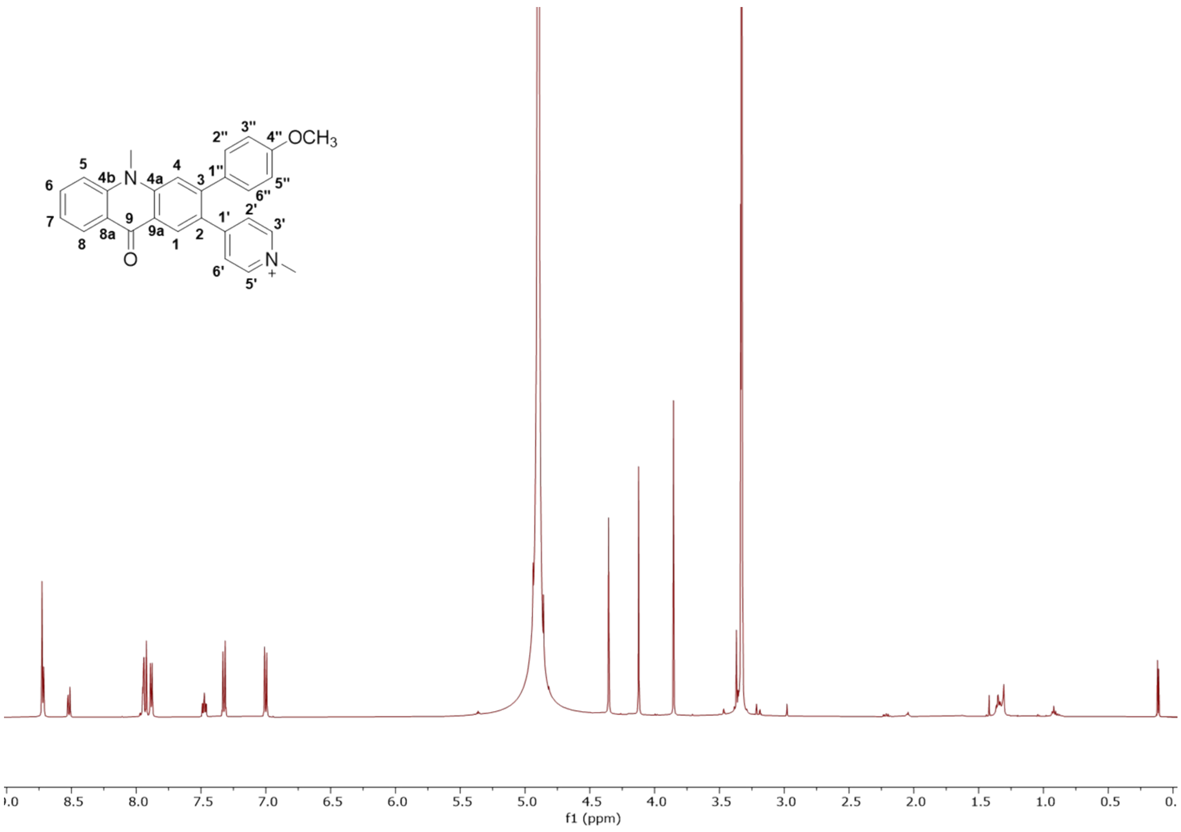

Figure S3. ^{1}H NMR spectrum of **AcridPyMe** (500.16 Hz, CD_3OD).

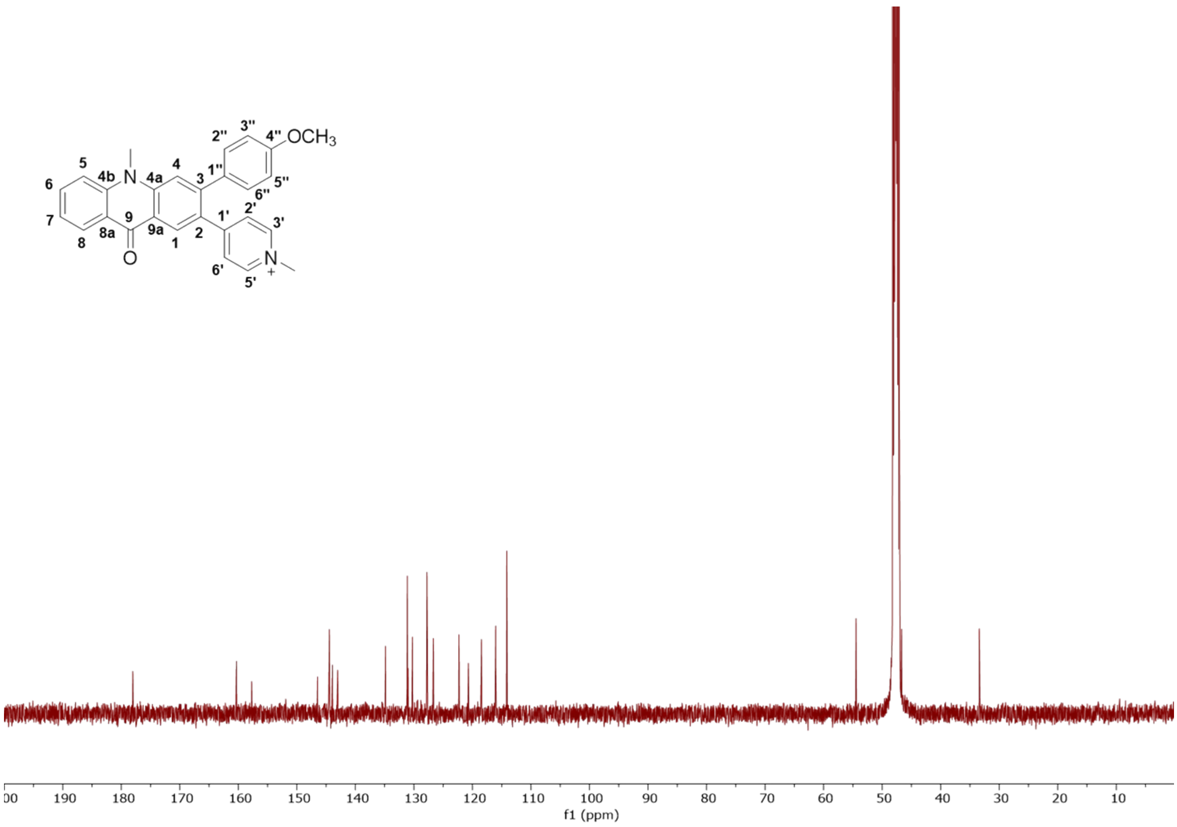

Figure S4. ^{13}C NMR spectrum of **AcridPyMe** (125.77 Hz, CD_3OD).

1.2. Mass Spectrometry Spectra

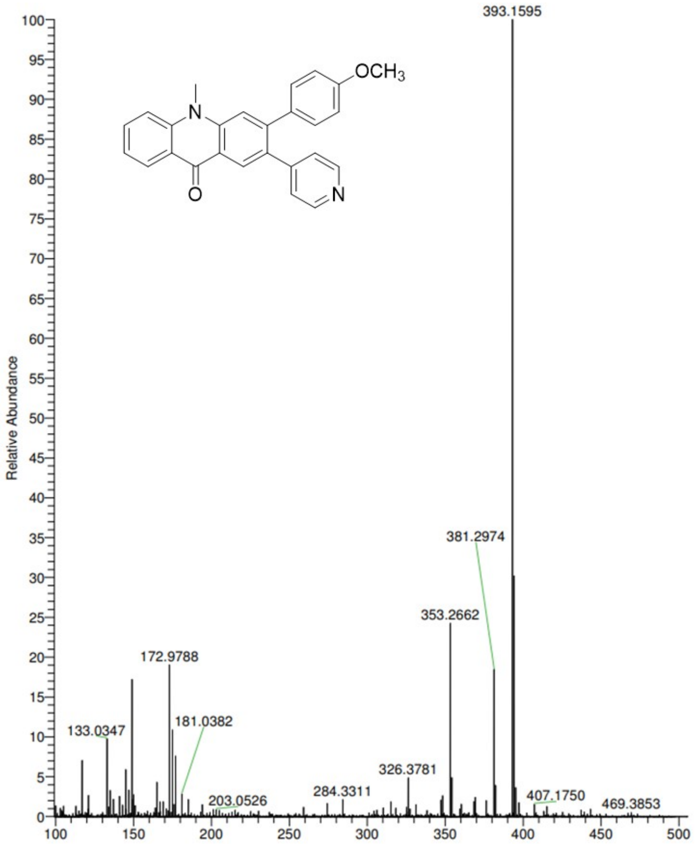

Figure S5. HRMS spectrum of **AcridPy**.

Table S1. AcridPy HRMS spectrum simulation.

m/z	Theoretical Mass	Delta (ppm)	RDB equiv.	Composition
393.1595	393.1598	-0.65	17.5	$C_{26}H_{21}O_2N_2$
	393.1589	1.46	0	$C_{11}H_{27}O_{12}N_3$
	393.1589	1.48	5.5	$C_{10}H_{21}O_7N_{10}$
	393.1603	-1.94	5	$C_{12}H_{23}O_8N_7$
	393.1603	-1.95	-0.5	$C_{13}H_{29}O_{13}$
	393.1584	2.77	18	$C_{24}H_{19}ON_5$
	393.1576	4.88	0.5	$C_9H_{25}O_{11}N_6$
	393.1616	-5.35	4.5	$C_{14}H_{25}O_9N_4$
	393.1571	6.17	13	$C_{23}H_{23}O_5N$
	393.1571	6.18	18.5	$C_{22}H_{17}N_8$

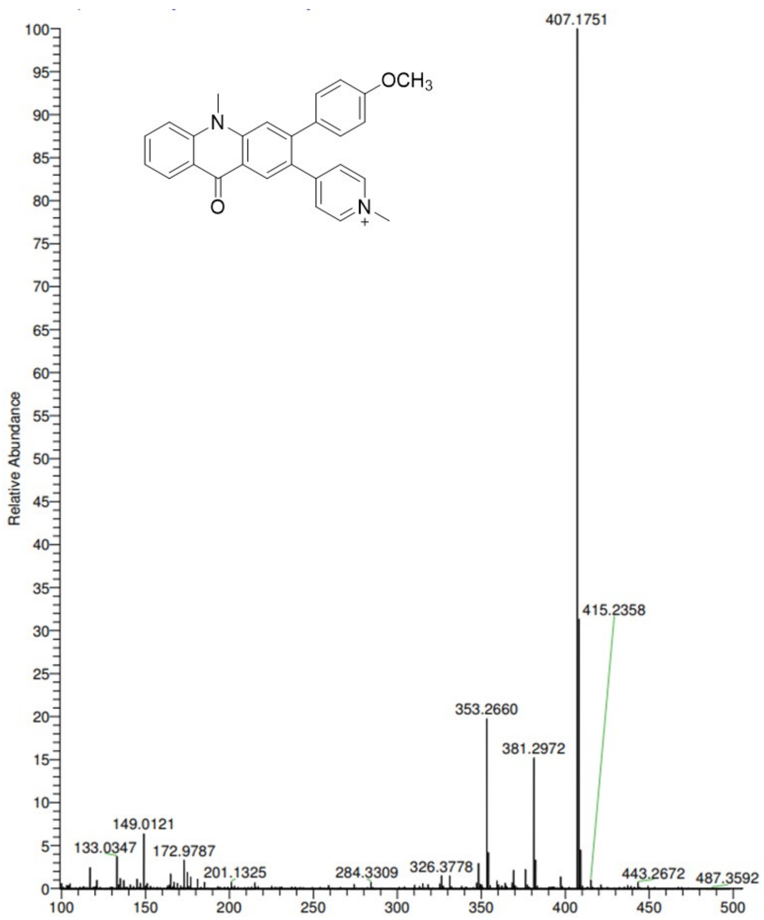

Figure S6. HRMS spectrum of **AcridPyMe**.

Table S2. AcridPyMe HRMS spectrum simulation.

m/z	Theoretical Mass	Delta (ppm)	RDB equiv.	Composition
407.1747	407.1746	0.22	18	$C_{25}H_{21}ON_5$
	407.1751	-1.03	5.5	$C_{11}H_{23}O_7N_{10}$
	407.1751	-1.04	0	$C_{12}H_{29}O_{12}N_3$
	407.1738	2.26	0.5	$C_{10}H_{27}O_{11}N_6$
	407.176	-3.08	17.5	$C_{27}H_{23}O_2N_2$
	407.1733	3.51	13	$C_{24}H_{25}O_5N$
	407.1733	3.52	18.5	$C_{23}H_{19}N_8$
	407.1765	-4.32	5	$C_{13}H_{25}O_8N_7$
	407.1765	-4.34	-0.5	$C_{14}H_{31}O_{13}$
	407.1724	5.56	1	$C_8H_{25}O_{10}N_9$

1.3. Molar Extinction Coefficient

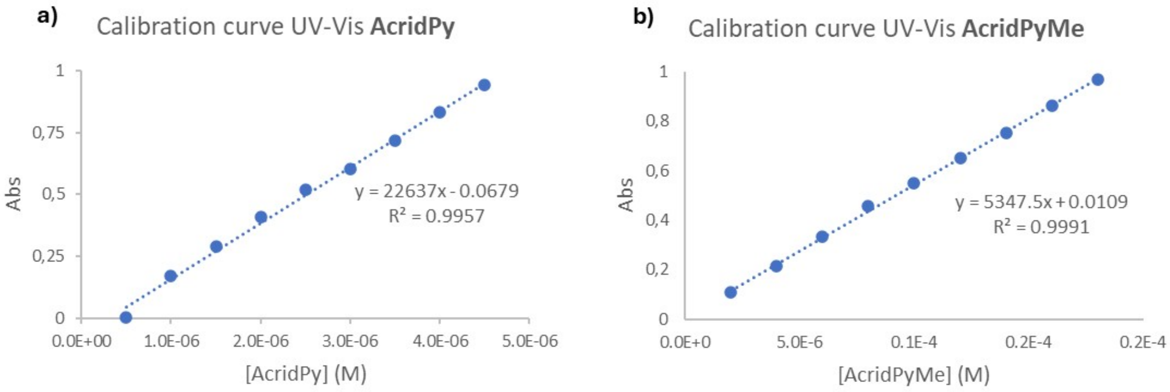

Figure S7. UV-Vis calibration curve for the determination of molar extinction coefficient of **a**) **AcridPy** and **b**) **AcridPyMe**.

1.4. HPLC/UV-Vis

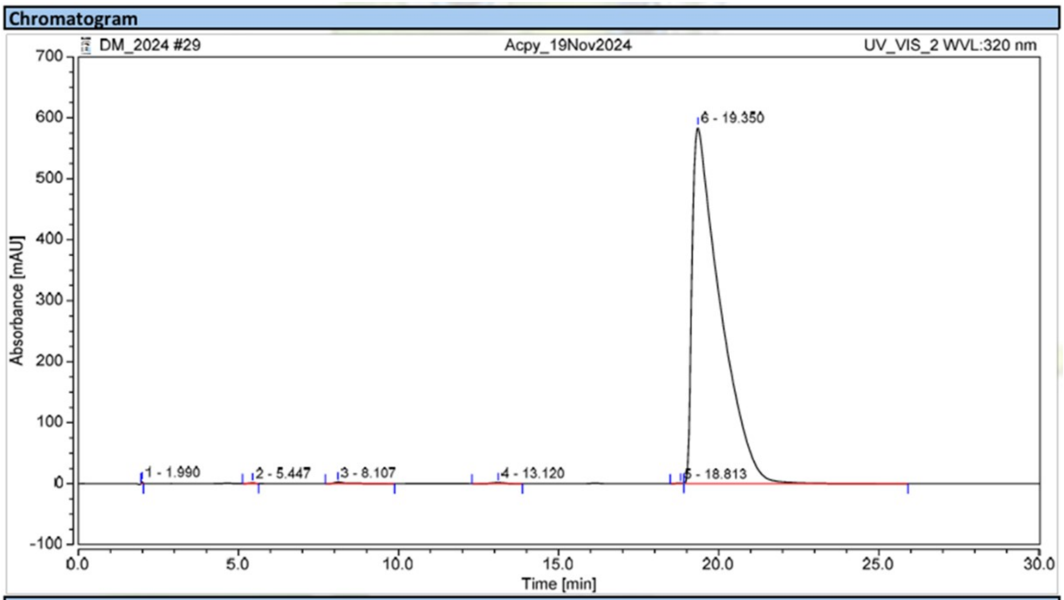

Integration Results

No.	Peak Name	Retention Time min	Area mAU*min	Height mAU	Relative Area %	Relative Height %	Amount n.a.
1		1.990	0.125	2.962	0.02	0.50	n.a.
2		5.447	0.245	1.303	0.04	0.22	n.a.
3		8.107	1.326	2.726	0.22	0.46	n.a.
4		13.120	1.016	2.099	0.17	0.35	n.a.
5		18.813	0.184	0.672	0.03	0.11	n.a.
6		19.350	591.605	584.841	99.51	98.36	n.a.
**Total:**			**594.501**	**594.603**	**100.00**	**100.00**	

Figure S8. HPLC/UV-Vis analysus of **AcridPy** with a detection wavelength of 320 nm.

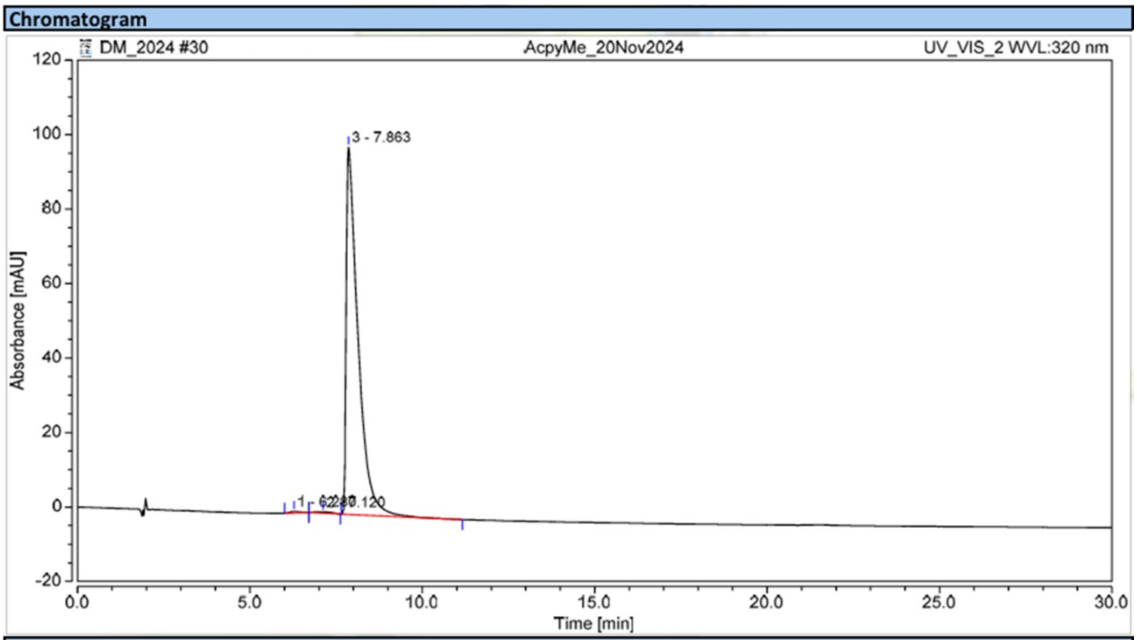

Integration Results

No.	Peak Name	Retention Time min	Area mAU*min	Height mAU	Relative Area %	Relative Height %	Amount n.a.
1		6.280	0.104	0.372	0.27	0.37	n.a.
2		7.120	0.161	0.288	0.42	0.29	n.a.
3		7.863	38.612	98.654	99.32	99.34	n.a.
Total:			38.877	99.314	100.00	100.00	

Figure S9. HPLC/UV-Vis analysus of **AcridPyMe** with a detection wavelength of 320 nm.

1.5. Fluorescence Quantum Yield

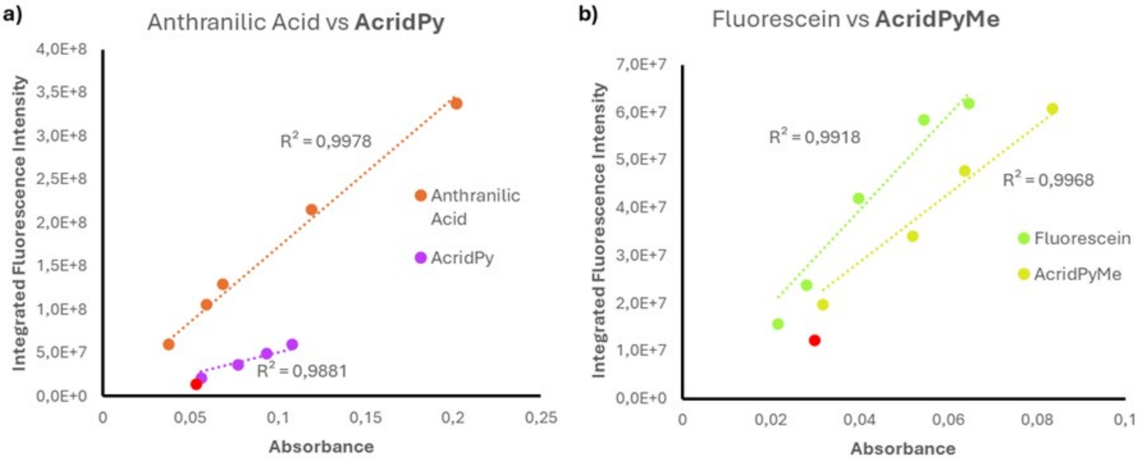

Figure S10. Calibration curves of **a**) anthranilic acid and **AcridPy** and **b**) fluorescein and **AcridPyMe**. The red dots are considered outliers and, therefore, were withdrawn from the calibration curves.

2. Fluorescence spectroscopy

2.1. Fluorescence titrations

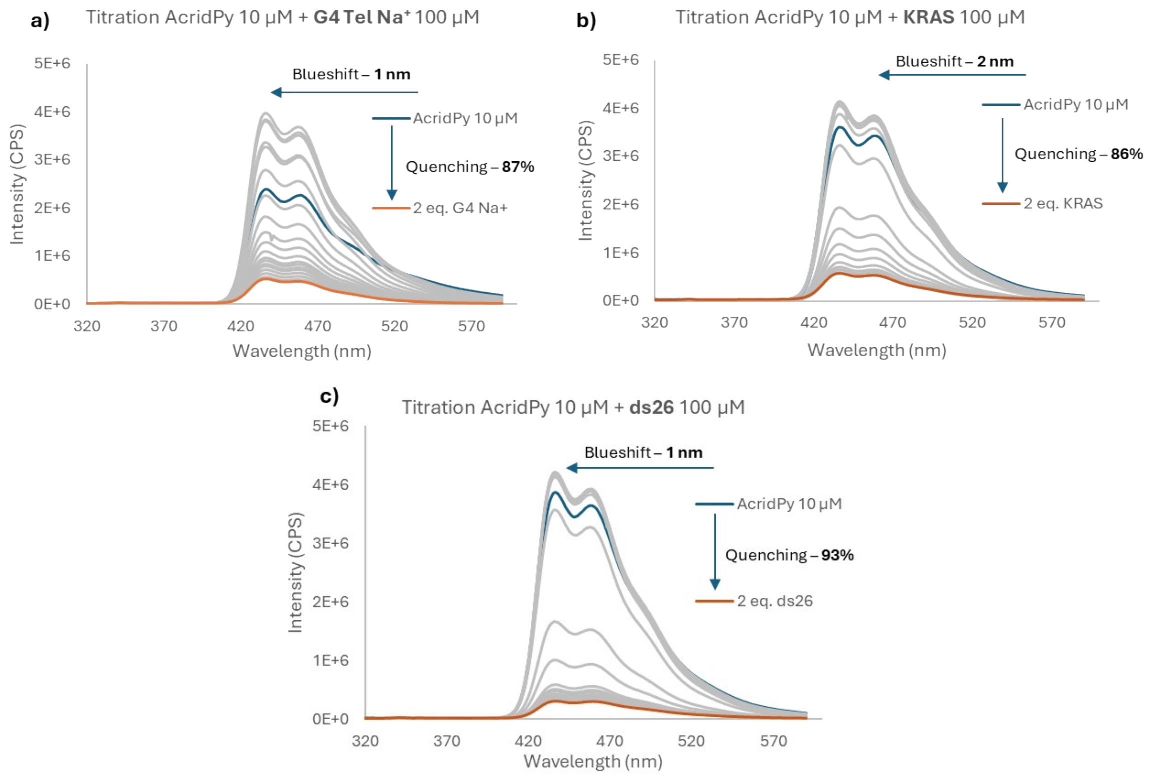

Figure S11. Fluorescence titrations spectra of **AcridPy** with **a**) telomeric G4 in Na^{+}, **b**) KRAS, and **c**) ds26.

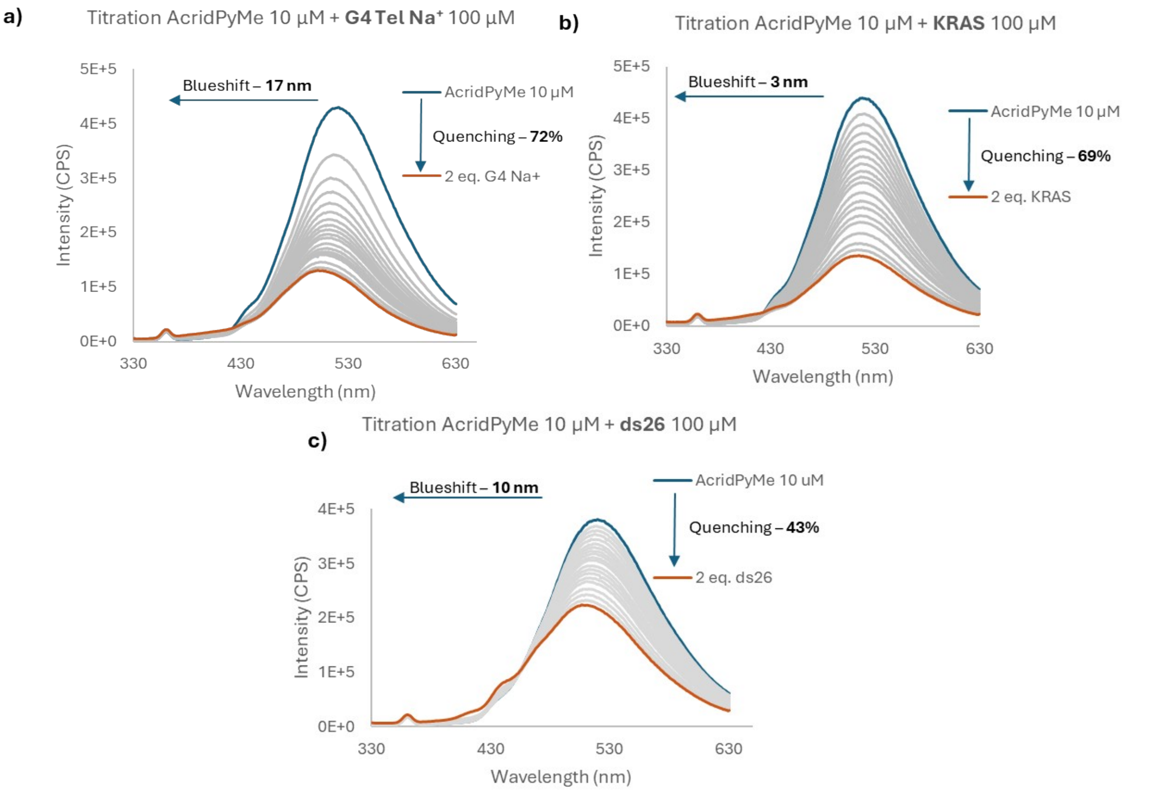

Figure S12. Fluorescence titrations spectra of **AcridPyMe** with **a**) telomeric G4 in Na^{+}, **b**) KRAS, and **c**) ds26.

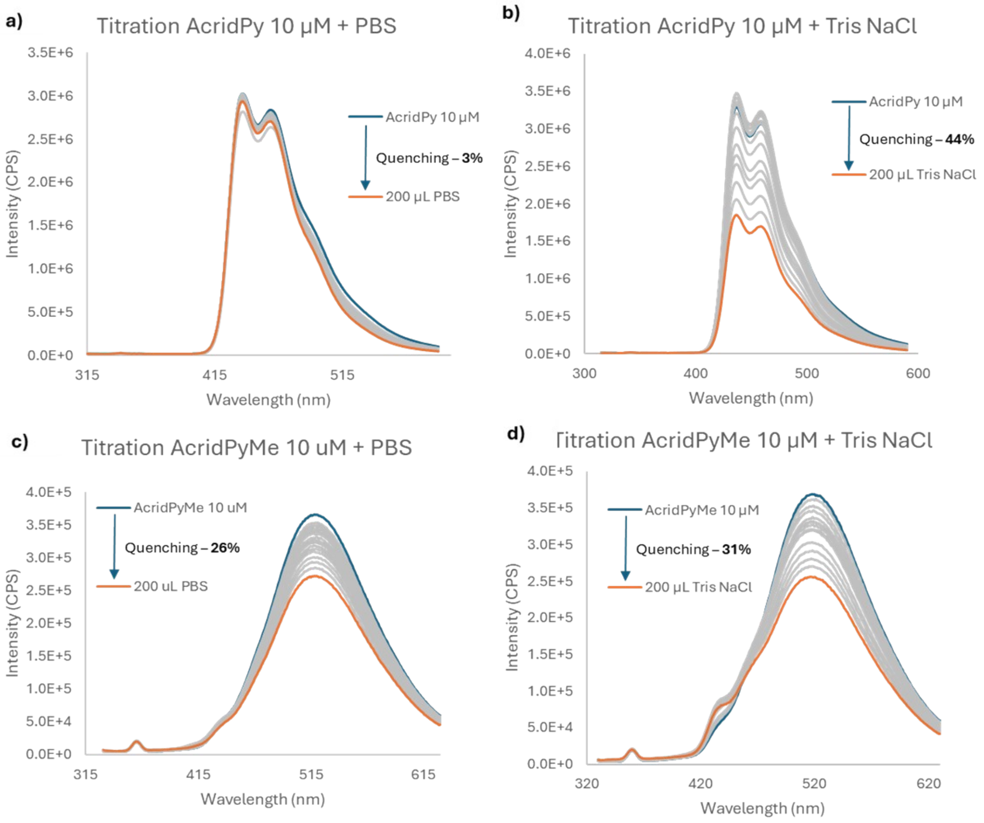

Figure S13. Fluorescence titrations of both **a) AcridPy** with PBS, **b) AcridPy** with Tris NaCl, **c) AcridPyMe** with PBS and **d) AcridPyMe** with Tris NaCl.

2.2. Time control spectra

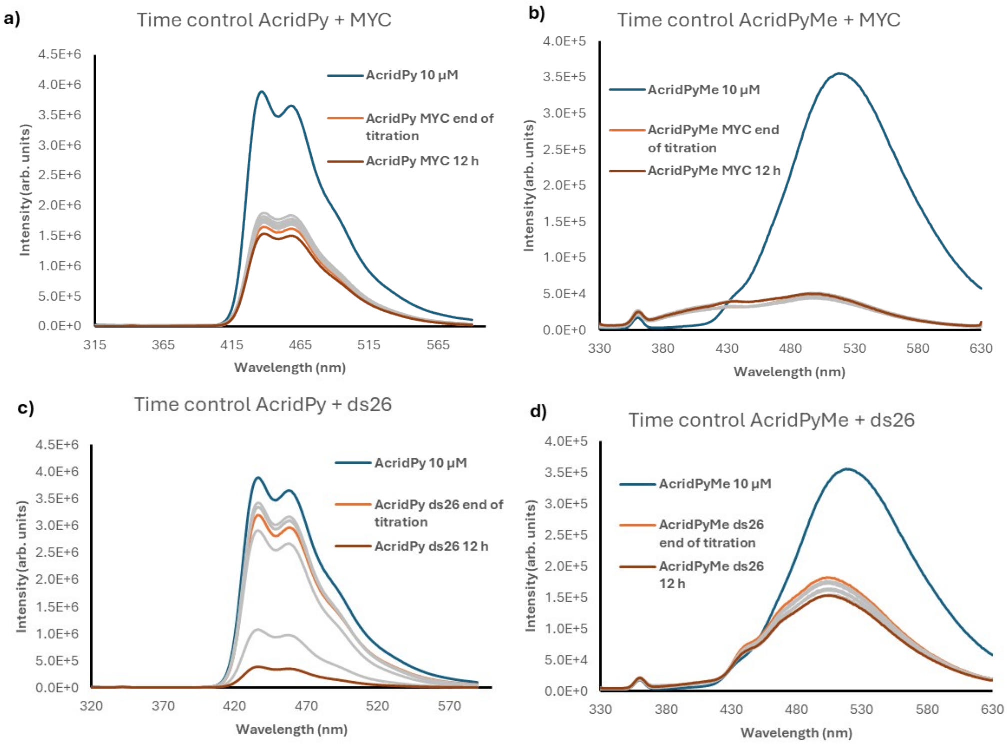

Figure S14. Fluorescence titrations spectra of MYC with **a**) **AcridPy** and **b**) **AcridPyMe** and ds26 with **c**) **AcridPy** and **d**) **AcridPyMe**.

2.3. FID Assay

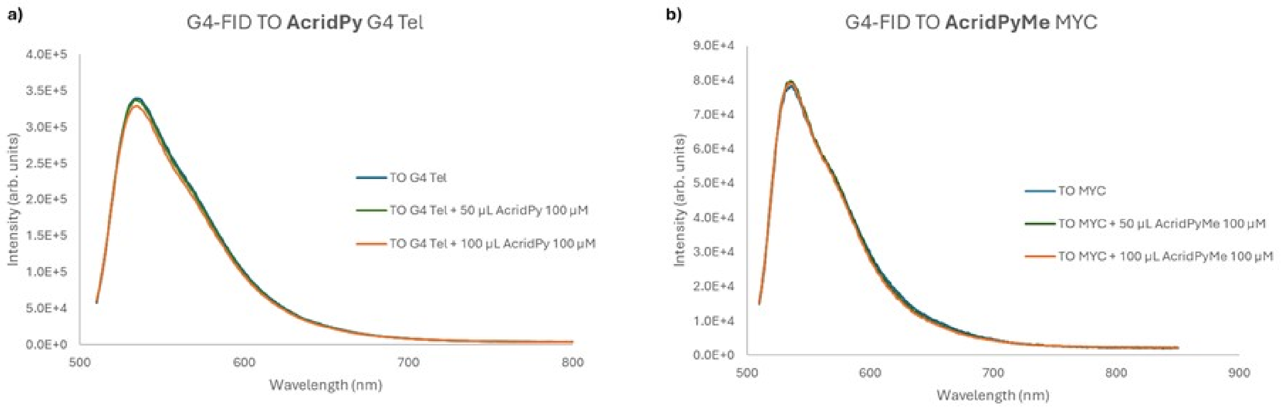

Figure S15. FID assay **a**) with G4 Tel-TO adduct and increasing amounts of **AcridPy** and **b**) with MYC-TO adduct and increasing amounts of **AcridPyMe**.

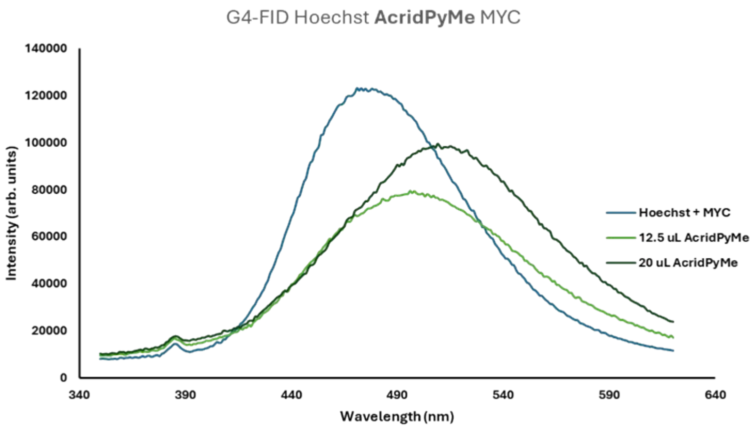

Figure S16. Fluorescent Intercalator Displacement assay with MYC-Hoechst adduct and increasing amounts of **AcridPyMe**.

3. Circular Dichroism

3.1. CD Spectra

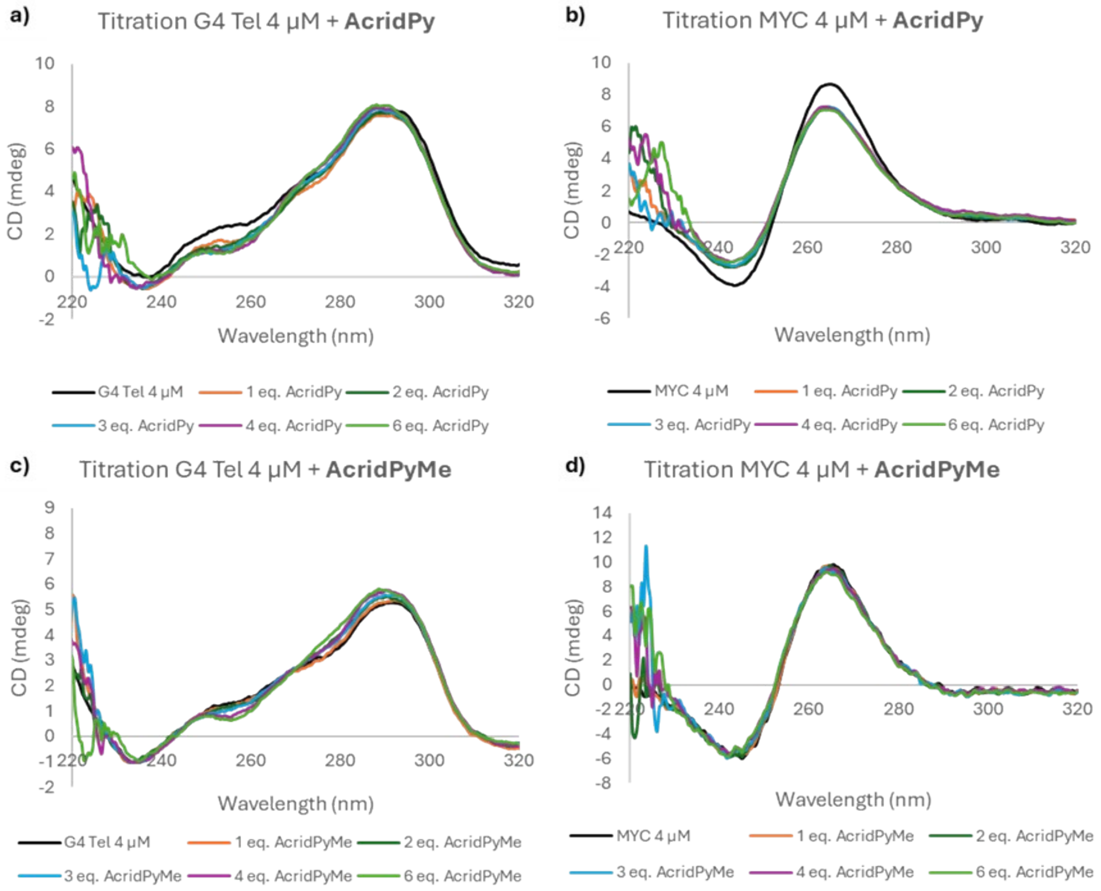

Figure S17. CD spectra of titrations of **a**) G4 Tel and **b**) MYC with **AcridPy**, and **c**) G4 Tel and **d**) MYC with **AcridPyMe**.

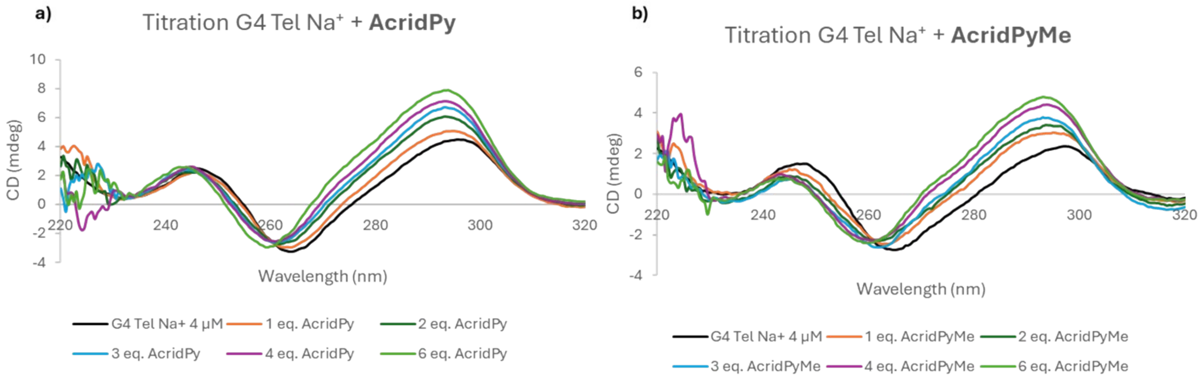

Figure S18. CD spectra of titrations of G4 Tel Na^+ with **a**) **AcridPy**, and **b**) **AcridPyMe**.

3.2. CD Melting

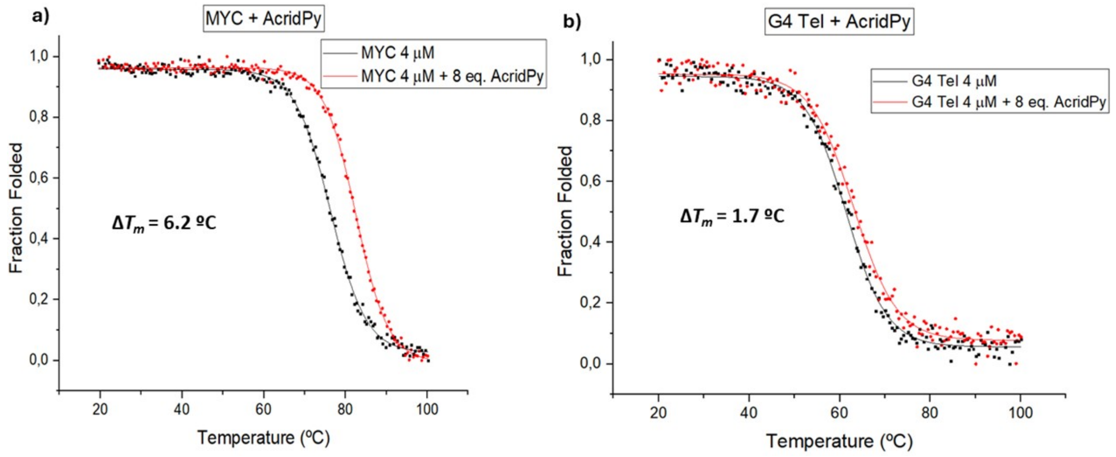

Figure S19. CD melting spectra **a**) MYC + **AcridPy**, and **b**) G4 Tel + **AcridPy**.

4. Molecular Dynamics

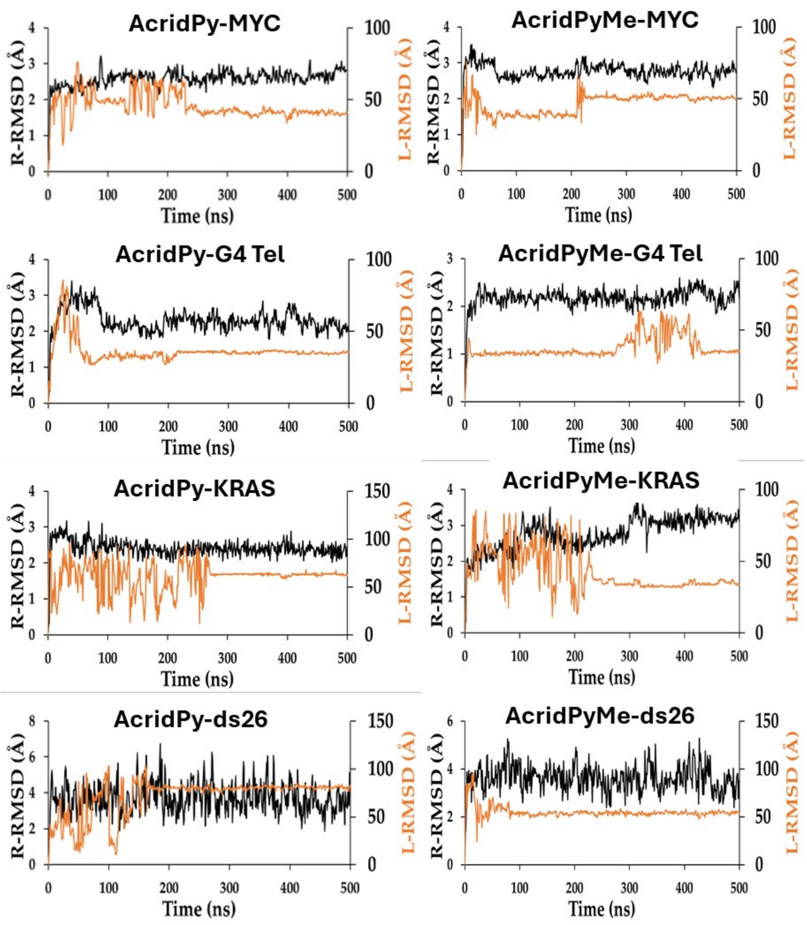

Figure S20. Receptor root mean-squared deviation (R-RMSD, black) and Ligand root mean-squared deviation (L-RMSD, orange) of each simulation system throughout the trajectory.

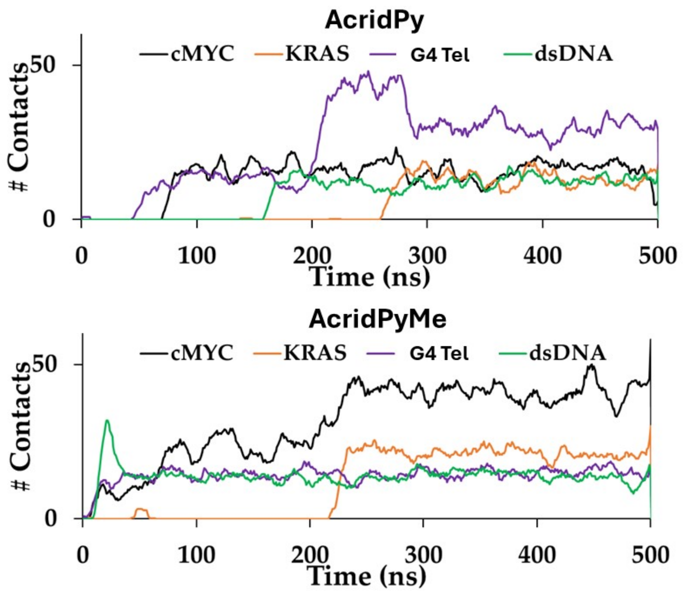

Figure S21. Atom contact map between compounds **AcridPy** and **AcridPyMe** and each nucleic acid structure; MYC, KRAS, G4 Tel and dsDNA are represented in black, orange, purple and green lines, respectively.

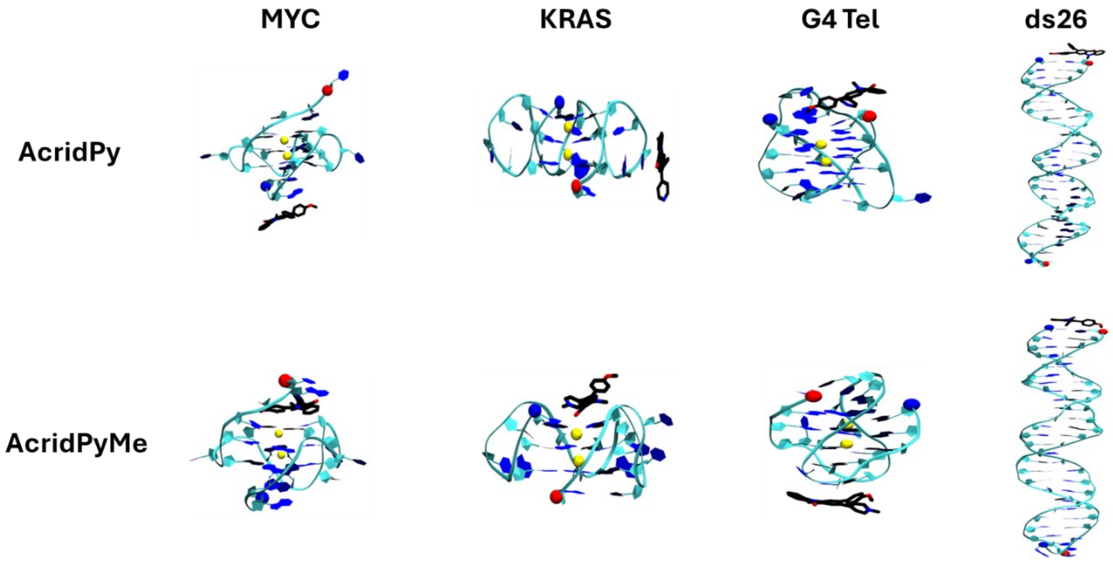

Figure S22. Last snapshot structures of **AcridPy** and **AcridPyMe** bound to MYC, KRAS, G4 Tel and ds26 structures.

Table S3. Ligand parameters of **AcridPy** and **AcridPyMe** in MOL2 format.

@<TRIPOS>ATOM

1	C1	5.7270	0.7520	-0.3000	ca	1 APY	-0.106014
2	H1	5.8870	1.8130	-0.3340	ha	1 APY	0.153662
3	C2	4.4220	0.3050	-0.0820	ca	1 APY	-0.036474
4	C3	4.1490	-1.0650	-0.0160	ca	1 APY	0.036856
5	C4	5.2100	-1.9630	-0.2100	ca	1 APY	-0.169423
6	H2	5.0400	-3.0210	-0.2200	ha	1 APY	0.126736

7	C5	6.4860	-1.4960	-0.4270	ca	1	APY	-0.126538
8	H3	7.2790	-2.2080	-0.5780	ha	1	APY	0.153799
9	C6	6.7640	-0.1320	-0.4660	ca	1	APY	-0.185827
10	H4	7.7660	0.2180	-0.6350	ha	1	APY	0.146887
11	C7	3.3430	1.2990	0.0530	c	1	APY	0.470637
12	C8	1.9860	0.7320	0.1080	ca	1	APY	-0.042557
13	C9	1.7840	-0.6470	0.1720	ca	1	APY	-0.055326
14	N1	2.8580	-1.5200	0.2290	na	1	APY	0.011318
15	C10	0.8890	1.5900	0.0850	ca	1	APY	-0.098465
16	H5	1.0960	2.6430	0.0720	ha	1	APY	0.137599
17	C11	-0.4130	1.1380	0.1090	cp	1	APY	-0.048748
18	C12	-0.6230	-0.2580	0.1280	cq	1	APY	0.030107
19	C13	0.4620	-1.1140	0.1580	ca	1	APY	-0.153582
20	H6	0.2650	-2.1660	0.1110	ha	1	APY	0.110918
21	C14	-1.9840	-0.8750	0.0720	cq	1	APY	0.012987
22	C15	-1.5150	2.1460	0.1410	cp	1	APY	0.187708
23	C16	-2.3850	-1.8020	1.0360	ca	1	APY	-0.067205
24	H7	-1.7230	-2.0410	1.8490	ha	1	APY	0.133571
25	C17	-3.6230	-2.4040	0.9810	ca	1	APY	-0.296987
26	H8	-3.9330	-3.1080	1.7300	ha	1	APY	0.157239
27	C18	-4.5020	-2.1010	-0.0560	ca	1	APY	0.310366
28	C19	-4.1190	-1.1890	-1.0270	ca	1	APY	-0.201898
29	H9	-4.7700	-0.9370	-1.8410	ha	1	APY	0.140915
30	C20	-2.8710	-0.5860	-0.9520	ca	1	APY	-0.175226
31	H10	-2.5930	0.1180	-1.7150	ha	1	APY	0.155977
32	C21	-1.5820	3.1580	-0.8090	ca	1	APY	-0.267131
33	H11	-0.8680	3.2090	-1.6100	ha	1	APY	0.121064
34	C22	-2.5910	4.1020	-0.7200	ca	1	APY	0.263511
35	H12	-2.6570	4.8890	-1.4510	h4	1	APY	0.069184
36	N2	-3.5120	4.1060	0.2250	nb	1	APY	-0.571887
37	C23	-3.4460	3.1470	1.1320	ca	1	APY	0.283327
38	H13	-4.2000	3.1690	1.9000	h4	1	APY	0.064467

39 C24	-2.4840	2.1550	1.1390 ca	1 APY	-0.266139
40 H14	-2.4880	1.4090	1.9110 ha	1 APY	0.116992
41 O1	3.5520	2.4840	0.0730 o	1 APY	-0.538328
42 O2	-5.6870	-2.7400	-0.0290 os	1 APY	-0.327376
43 C25	-6.6420	-2.4770	-1.0180 c3	1 APY	0.014050
44 H15	-7.4990	-3.0880	-0.7780 h1	1 APY	0.065758
45 H16	-6.9360	-1.4330	-1.0160 h1	1 APY	0.065758
46 H17	-6.2770	-2.7460	-2.0030 h1	1 APY	0.065758
47 C26	2.6420	-2.9100	0.5950 c3	1 APY	-0.086063
48 H18	1.7890	-2.9830	1.2500 h1	1 APY	0.071347
49 H19	2.4780	-3.5450	-0.2710 h1	1 APY	0.071347
50 H20	3.4950	-3.2770	1.1430 h1	1 APY	0.071347

@<TRIPOS>ATOM

1 C1	-5.8330	1.0560	0.3290 ca	1 APM	-0.114821
2 H1	-5.9000	2.1230	0.4230 ha	1 APM	0.164966
3 C2	-4.5700	0.5030	0.1020 ca	1 APM	-0.012987
4 C3	-4.4210	-0.8800	-0.0370 ca	1 APM	0.033076
5 C4	-5.5560	-1.6880	0.0950 ca	1 APM	-0.173635
6 H2	-5.4840	-2.7550	0.0450 ha	1 APM	0.139036
7 C5	-6.7900	-1.1190	0.3240 ca	1 APM	-0.106155
8 H3	-7.6450	-1.7630	0.4260 ha	1 APM	0.163543
9 C6	-6.9450	0.2600	0.4340 ca	1 APM	-0.176440
10 H4	-7.9140	0.6870	0.6100 ha	1 APM	0.160510
11 C7	-3.4040	1.3930	0.0460 c	1 APM	0.438578
12 C8	-2.1050	0.6980	-0.0350 ca	1 APM	-0.033311
13 C9	-2.0280	-0.6910	-0.1870 ca	1 APM	-0.013992
14 N1	-3.1640	-1.4450	-0.2950 na	1 APM	0.008705
15 C10	-0.9450	1.4490	0.0370 ca	1 APM	-0.076017
16 H5	-1.0760	2.5130	0.1000 ha	1 APM	0.146989
17 C11	0.3180	0.8850	-0.0290 ca	1 APM	-0.041996
18 C12	0.4090	-0.5290	-0.1260 cp	1 APM	-0.011356
19 C13	-0.7430	-1.2770	-0.2000 ca	1 APM	-0.128490

20 H6	-0.6410	-2.3420	-0.2010 ha	1 APM	0.135221
21 C14	1.7100	-1.2620	-0.0730 cp	1 APM	-0.001057
22 C15	1.4730	1.7970	-0.0650 cc	1 APM	0.105809
23 C16	2.0930	-2.1140	-1.1120 ca	1 APM	-0.049514
24 H7	1.4590	-2.2210	-1.9740 ha	1 APM	0.132755
25 C17	3.2770	-2.8140	-1.0580 ca	1 APM	-0.295186
26 H8	3.5740	-3.4650	-1.8580 ha	1 APM	0.169594
27 C18	4.1130	-2.6980	0.0520 ca	1 APM	0.344125
28 C19	3.7420	-1.8640	1.0980 ca	1 APM	-0.214923
29 H9	4.3530	-1.7670	1.9740 ha	1 APM	0.145410
30 C20	2.5500	-1.1560	1.0240 ca	1 APM	-0.194837
31 H10	2.2700	-0.5310	1.8530 ha	1 APM	0.168621
32 C21	1.4930	2.9590	0.7210 cc	1 APM	-0.128066
33 H11	0.7060	3.1660	1.4190 ha	1 APM	0.160868
34 C22	2.5320	3.8420	0.6300 cd	1 APM	-0.054513
35 H12	2.5680	4.7330	1.2250 h4	1 APM	0.197909
36 N2	3.5590	3.6330	-0.2010 na	1 APM	0.103171
37 C23	3.5750	2.5290	-0.9670 cd	1 APM	-0.056097
38 H13	4.4150	2.4200	-1.6240 h4	1 APM	0.190995
39 C24	2.5730	1.6070	-0.9210 cc	1 APM	-0.098395
40 H14	2.6330	0.7520	-1.5620 ha	1 APM	0.144255
41 O1	-3.4770	2.5920	0.1020 o	1 APM	-0.527544
42 O2	5.2390	-3.4210	0.0140 os	1 APM	-0.328826
43 C25	6.1040	-3.4470	1.1200 c3	1 APM	-0.001392
44 H15	6.9020	-4.1220	0.8590 h1	1 APM	0.077413
45 H16	6.5170	-2.4640	1.3190 h1	1 APM	0.077413
46 H17	5.5970	-3.8150	2.0050 h1	1 APM	0.077413
47 C26	-3.0840	-2.8380	-0.7200 c3	1 APM	-0.085695
48 H18	-2.2210	-2.9760	-1.3510 h1	1 APM	0.078275
49 H19	-3.0250	-3.5190	0.1220 h1	1 APM	0.078275
50 H20	-3.9500	-3.0800	-1.3140 h1	1 APM	0.078275
51 C27	4.6640	4.6030	-0.3080 c3	1 APM	-0.181864

52 H21	5.6030	4.0940	-0.1510 h1	1 APM	0.128636
53 H22	4.6480	5.0600	-1.2870 h1	1 APM	0.128636
54 H23	4.5390	5.3630	0.4460 h1	1 APM	0.128636

5. Biological Assays

5.1. MTT Assay

Table S4. MTT assay results of **AcridPyMe** effect on the cell viability of PanC-1 cell line. Data is represented by mean values ± standard deviation of three independent experiments with three technical replicates each.

	PanC-1		
**Concentration (µM)**	**24 h**	**48 h**	**72 h**
**0**	100 ± 4.34	100 ± 5.26	100 ± 5.34
**0.5**	93.5 ± 5.08	92.4 ± 9.30	94.1 ± 6.68
**1**	94.5 ± 7.75	82.1 ± 5.18	91.1 ± 10.0
**2.5**	96.4 ± 7.65	89.5 ± 8.72	90.6 ± 10.2
**5**	95.7 ± 6.38	81.5 ± 5.47	88.3 ± 13.1
**10**	94.5 ± 9.77	78.7 ± 6.26	86.3 ± 11.2
**25**	90.6 ± 4.91	78.3 ± 6.99	80.8 ± 12.7
**50**	84.9 ± 8.46	74.4 ± 6.22	80.5 ± 12.6
**75**	89.0 ± 6.53	71.9 ± 6.59	68.8 ± 6.60
**100**	76.4 ± 6.27	65.0 ± 5.57	69.1 ± 3.31

Table S5. MTT assay results of **AcridPyMe** effect on the cell viability of MIA PaCa-2 cell line. Data is represented by mean values ± standard deviation of three independent experiments with three technical replicates each.

	MIA PaCa-2		
**Concentration (µM)**	**24 H**	**48 H**	**72 H**
**0**	100 ± 3.64	100 ± 5.42	100 ± 6.29
**0.5**	96.3 ± 7.01	93.7 ± 9.90	95.9 ± 8.11
**1**	99.9 ± 10.4	90.5 ± 7.89	94.4 ± 7.76
**2.5**	100.5 ± 3.14	94.8 ± 7.94	93.1 ± 5.37
**5**	99.4 ± 8.06	90.5 ± 6.94	97.1 ± 6.31

**10**	95.5 ± 6.14	89.1 ± 8.5	89.2 ± 8.11
**25**	100.1 ± 6.28	87.7 ± 9.95	77.9 ± 8.12
**50**	94.1 ± 5.62	74.4 ± 7.12	62.8 ± 7.30
**75**	94.9 ± 10.2	65.7 ± 5.43	47.6 ± 9.95
**100**	84.3 ± 12.3	58.4 ± 9.33	35.9 ± 7.39

Table S6. MTT assay results of **AcridPyMe** effect on the cell viability of A549 cell line. Data is represented by mean values ± standard deviation of three independent experiments with three technical replicates each.

	**A549**		
**Concentration (µM)**	**24 h**	**48 h**	**72 h**
**0**	100 ± 5.93	100 ± 3.37	100 ± 3.55
**0.5**	107 ± 9.05	103 ± 4.07	103 ± 6.28
**1**	105 ± 7.80	104 ± 7.08	98.3 ± 7.07
**2.5**	104 ± 7.55	100 ± 6.34	91.9 ± 6.37
**5**	98.1 ± 8.66	102 ± 6.91	92.5 ± 9.40
**10**	98.9 ± 7.85	99.4 ± 4.53	92.7 ± 8.76
**25**	103 ± 7.74	97.4 ± 3.39	91.7 ± 8.54
**50**	102 ± 6.57	96.7 ± 3.24	90.3 ± 4.59
**75**	102 ± 9.30	97.0 ± 3.62	90.7 ± 7.41
**100**	98.7 ± 7.88	92.0 ± 8.30	84.4 ± 6.07

Table S7. MTT assay results of **AcridPyMe** effect on the cell viability of A375 cell line. Data is represented by mean values ± standard deviation of three independent experiments with three technical replicates each.

	**A375**		
**Concentration (µM)**	**24 h**	**48 h**	**72 h**
**0**	100 ± 5.43	100 ± 2.72	100 ± 4.54
**0.5**	114 ± 7.71	101 ± 6.48	97.5 ± 8.16
**1**	113 ± 7.48	98.0 ± 6.96	95.8 ± 8.83
**2.5**	110 ± 6.67	95.6 ± 6.67	87.4 ± 5.37

**5**	108 ± 7.36	93.7 ± 7.04	88.9 ± 5.09
**10**	112 ± 9.27	93.1 ± 9.99	89.0 ± 4.67
**25**	110 ± 7.83	94.9 ± 7.13	87.8 ± 7.87
**50**	115 ± 7.67	92.8 ± 9.41	91.1 ± 6.66
**75**	113 ± 6.41	96.5 ± 5.62	92.2 ± 8.70
**100**	109 ± 7.92	95.8 ± 6.57	95.1 ± 10.1

Table S8. MTT assay results of **AcridPyMe** effect on the cell viability of HaCaT cell line. Data is represented by mean values ± standard deviation of three independent experiments with three technical replicates each.

	**HaCaT**		
**Concentration (µM)**	**24 h**	**48 h**	**72 h**
**0**	100 ± 7.61	100 ± 8.03	100 ± 4.70
**0.5**	115 ± 8.20	99.4 ± 8.67	106 ± 8.03
**1**	117 ± 9.94	98.9 ± 8.91	100 ± 8.89
**2.5**	112 ± 10.1	97.2 ± 11.0	95.7 ± 6.65
**5**	117 ± 10.9	92.5 ± 9.27	94.3 ± 7.38
**10**	119 ± 10.7	99.1 ± 7.61	89.9 ± 6.40
**25**	112 ± 9.65	92.6 ± 8.36	94.8 ± 5.49
**50**	117 ± 10.4	88.6 ± 10.9	92.1 ± 6.49
**75**	113 ± 12.2	94.1 ± 10.7	94.4 ± 6.05
**100**	102 ± 9.05	93.9 ± 7.36	90.9 ± 6.51

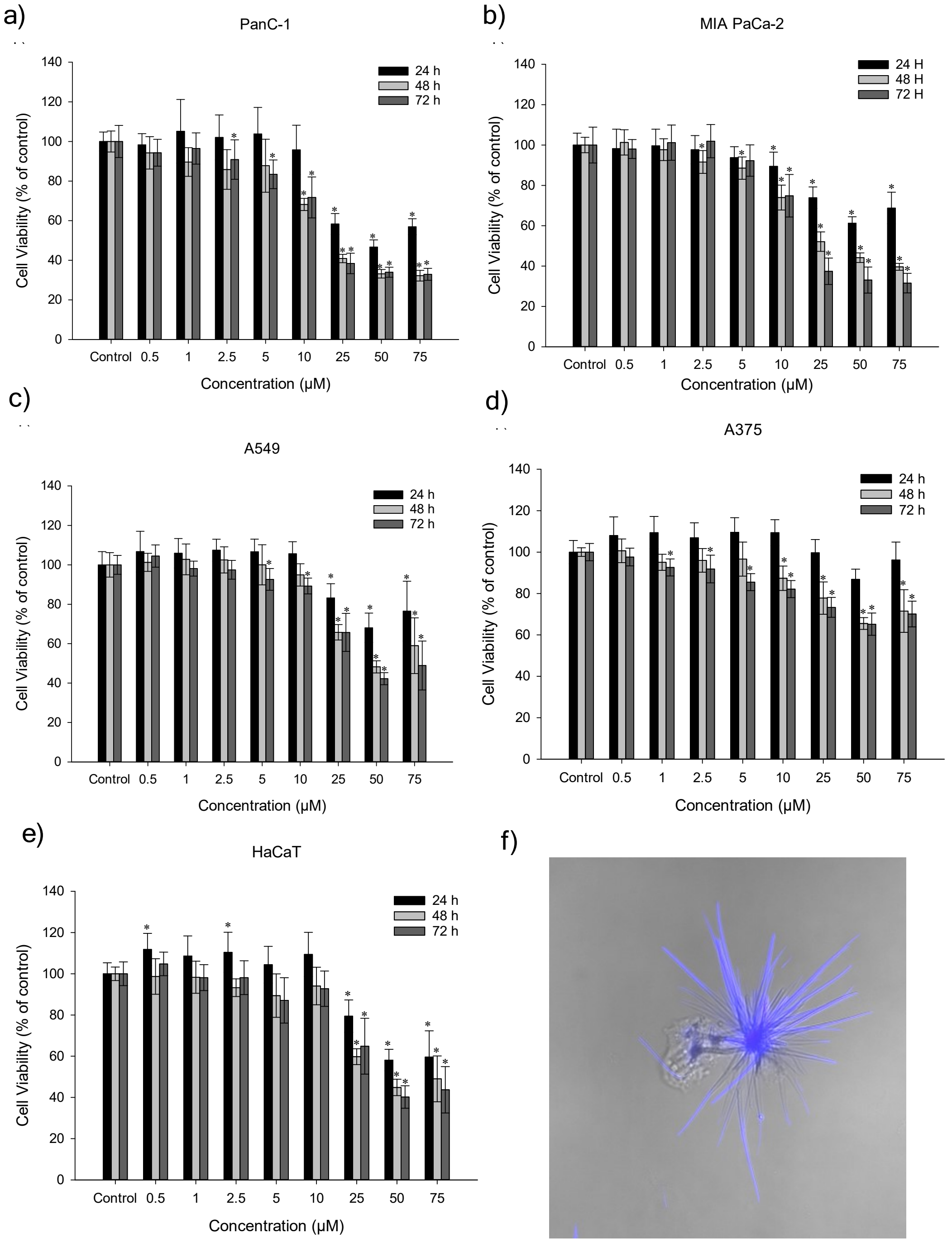

Figure S23. MTT assay to assess **AcridPy** effect on the cell viability of **a**) PanC-1, **b**) MIA PaCa-2, **c**) A549, **d**) A375, **e**) HaCaT cell lines. Data is represented by mean values ± standard deviation of three independent experiments with three technical replicates each. "*" is indicative of statistical significance in comparison to control ($p < 0.05$). **f**) Crystal-like aggregates formed by **AcridPy** when incubated with MIA PaCa-2 cells in cellular medium. The image was captured using a Zeiss LSM 880LSM 510 META confocal microscope (Zeiss, Jena, Germany) equipped with a Plan-Neofluor 63×/1.4 oil immersion objective.

Table S9. MTT assay results of **AcridPy** effect on the cell viability of PanC-1 cell line. Data is represented by mean values ± standard deviation of three independent experiments with three technical replicates each.

	PanC-1		
**Concentration (µM)**	**24 h**	**48 h**	**72 h**
**0**	100 ± 4.79	100 ± 5.30	100 ± 8.10
**0.5**	98.3 ± 5.69	94.3 ± 8.2	94.3 ± 6.79
**1**	105 ± 16.1	89.6 ± 7.23	96.5 ± 7.86
**2.5**	102 ± 13.3	85.9 ± 10.0	90.9 ± 9.96
**5**	104 ± 13.3	87.8 ± 13.4	83.4 ± 7.23
**10**	95.8 ± 12.5	68.2 ± 3.06	71.8 ± 10.4
**25**	58.3 ± 5.30	40.9 ± 2.11	38.4 ± 5.16
**50**	46.7 ± 3.59	33.2 ± 2.16	33.9 ± 2.53
**75**	56.9 ± 4.04	32.2 ± 2.69	32.9 ± 3.03

Table S10. MTT assay results of **AcridPy** effect on the cell viability of MIA PaCa-2 cell line. Data is represented by mean values ± standard deviation of three independent experiments with three technical replicates each.

	MIA PaCa-2		
**Concentration (µM)**	**24 h**	**48 h**	**72 h**
**0**	100 ± 5.86	100 ± 3.82	100 ± 8.89
**0.5**	98.2 ± 10.1	101 ± 6.26	97.9 ± 5.86
**1**	99.6 ± 8.26	97.7 ± 5.39	101 ± 8.74
**2.5**	97.7 ± 7.00	91.6 ± 5.64	102 ± 8.23
**5**	93.7 ± 5.44	88.6 ± 5.54	92.3 ± 7.76
**10**	89.4 ± 7.02	74.0 ± 6.17	74.9 ± 10.5
**25**	73.9 ± 5.31	52.1 ± 4.82	37.4 ± 6.53
**50**	61.2 ± 3.26	44.2 ± 2.36	33.1 ± 6.47
**75**	68.8 ± 3.26	39.6 ± 1.71	31.5 ± 4.87

Table S11. MTT assay results of **AcridPy** effect on the cell viability of A549 cell line. Data is represented by mean values ± standard deviation of three independent experiments with three technical replicates each.

	A549		
**Concentration (µM)**	**24 h**	**48 h**	**72 h**
**0**	100 ± 6.71	100 ± 6.20	100 ± 4.77
**0.5**	107 ± 10.3	101 ± 4.59	104 ± 5.65
**1**	106 ± 7.46	103 ± 7.80	98.1 ± 3.81
**2.5**	107 ± 5.57	103 ± 6.61	97.5 ± 4.76
**5**	107 ± 6.40	100 ± 10.2	92.6 ± 5.51
**10**	106 ± 6.15	94.9 ± 5.67	89.3 ± 4.02
**25**	83.2 ± 7.20	65.8 ± 3.92	65.7 ± 9.65
**50**	68.1 ± 7.43	48.2 ± 3.08	42.2 ± 3.04
**75**	76.5 ± 15.2	59.0 ± 14.8	48.9 ± 12.4

Table S12. MTT assay results of **AcridPy** effect on the cell viability of A375 cell line. Data is represented by mean values ± standard deviation of three independent experiments with three technical replicates each.

	A375		
**Concentration (µM)**	**24 h**	**48 h**	**72 h**
**0**	100 ± 5.62	100 ± 2.07	100 ± 4.18
**0.5**	108 ± 9.01	101 ± 5.70	97.6 ± 4.28
**1**	109 ± 7.80	95.1 ± 3.85	92.6 ± 4.10
**2.5**	107 ± 7.24	96.0 ± 5.71	91.8 ± 6.67
**5**	110 ± 7.08	96.6 ± 8.22	85.5 ± 8.07
**10**	109 ± 6.27	87.4 ± 5.90	82.1 ± 7.17
**25**	99.7 ± 6.41	77.8 ± 7.81	73.3 ± 4.80
**50**	86.9 ± 4.89	65.5 ± 2.85	65.2 ± 5.40
**75**	96.2 ± 8.68	71.5 ± 10.3	70.1 ± 6.20

Table S13. MTT assay results of **AcridPy** effect on the cell viability of HaCaT cell line. Data is represented by mean values ± standard deviation of three independent experiments with three technical replicates each.

	HaCaT		
**Concentration (µM)**	**24 h**	**48 h**	**72 h**
**0**	100 ± 5.31	100 ± 3.28	100 ± 5.75
**0.5**	112 ± 7.78	98.7 ± 8.61	105 ± 5.71
**1**	109 ± 9.70	98.3 ± 7.77	98.1 ± 6.28
**2.5**	110 ± 9.72	93.2 ± 4.28	98.1 ± 8.19
**5**	104 ± 8.92	89.4 ± 10.5	87.1 ± 11.0
**10**	109 ± 10.7	94.1 ± 9.12	92.8 ± 8.56
**25**	79.5 ± 7.83	59.8 ± 3.85	55.2 ± 13.6
**50**	58.1 ± 5.23	44.8 ± 3.98	40.2 ± 5.46
**75**	59.6 ± 12.8	49.0 ± 11.1	43.7 ± 11.2

5.2. Cell Cyle Analysis

Table S14. Effect of **AcridPyMe** on cell cycle distribution of MIA PaCa-2 cells. Data is represented by mean values ± standard deviation of two independent experiments with three technical replicates each and each replicate with at least 5000 events.

	**Control**	**AcridPyMe**
**G0/G1**	48.9 ± 3.58	53.9 ± 1.88
**S**	36.0 ± 2.56	37.1 ± 6.45
**G2/M**	15.1 ± 1.51	9.04 ± 5.81

5.3. Cell Apoptosis Assay

Table S15. Effect of **AcridPyMe** on apoptosis of MIA PaCa-2 cells. Data is represented by mean values ± standard deviation of two independent experiments with three technical replicates each and each replicate with at least 5000 events.

	**Control**	**AcridPyMe**
**Viable cells**	94.5 ± 2.50	88.7 ± 4.83
**Late apoptosis**	3.54 ± 2.06	3.85 ± 3.49
**Early apoptosis**	1.77 ± 0.69	5.95 ± 2.40
**Necrosis**	0.23 ± 0.06	1.48 ± 1.46
